# Distribution, classification, domain architectures and evolution of prolyl oligopeptidases in prokaryotic lineages

**DOI:** 10.1186/1471-2164-15-985

**Published:** 2014-11-18

**Authors:** Swati Kaushik, Ramanathan Sowdhamini

**Affiliations:** National Centre for Biological Sciences, Tata Institute of Fundamental Research, GKVK Campus, Bellary Road, Bangalore, 560065 India; Helen Diller Family Comprehensive Cancer Center, University of California, San Francisco, CA 94158 USA

**Keywords:** Prolyl oligopeptidase, Genome wide survey, Phylogeny, Bacterial prolyl oligopeptidase

## Abstract

**Background:**

Prolyl oligopeptidases (POPs) are proteolytic enzymes, widely distributed in all the kingdoms of life. Bacterial POPs are pharmaceutically important enzymes, yet their functional and evolutionary details are not fully explored. Therefore, current analysis is aimed at understanding the distribution, domain architecture, probable biological functions and gene family expansion of POPs in bacterial and archaeal lineages.

**Results:**

Exhaustive sequence analysis of 1,202 bacterial and 91 archaeal genomes revealed ~3,000 POP homologs, with only 638 annotated POPs. We observed wide distribution of POPs in all the analysed bacterial lineages. Phylogenetic analysis and co-clustering of POPs of different phyla suggested their common functions in all the prokaryotic species. Further, on the basis of unique sequence motifs we could classify bacterial POPs into eight subtypes. Analysis of coexisting domains in POPs highlighted their involvement in protein-protein interactions and cellular signaling. We proposed significant extension of this gene family by characterizing 39 new POPs and 158 new α/β hydrolase members.

**Conclusions:**

Our study reflects diversity and functional importance of POPs in bacterial species. Many genomes with multiple POPs were identified with high sequence variations and different cellular localizations. Such anomalous distribution of POP genes in different bacterial genomes shows differential expansion of POP gene family primarily by multiple horizontal gene transfer events.

**Electronic supplementary material:**

The online version of this article (doi:10.1186/1471-2164-15-985) contains supplementary material, which is available to authorized users.

## Background

Proteases are degradation machines that aid in the proper functioning of cells by sustaining the balance between protein synthesis and hydrolysis. The proteolytic enzymes are also involved in post-translational modifications and generation of active peptide molecules in the cell [1]. In fact, almost 2% of all proteins in the cell are proteolytic in nature, highlighting the decisive role of proteolytic enzymes in cellular regulatory circuits [2, 3]. Serine proteases are family of proteases that can cleave a peptide bond using a nucleophilic serine residue in their catalytic triad. They are involved in diverse biological processes, and are considered as attractive targets for drug design [4, 5]. Prokaryotic serine proteases play an important role in cell signaling, metabolism and various defense responses [1, 6, 7], and help these microorganisms to adapt to a wide variety of environments and growth conditions [8, 9].

A particular type of serine protease that can hydrolyse internal proline residues distinctly is referred as prolyl oligopeptidase (POP, family S9A, according to MEROPS database) [2, 10, 11]. POPs are distinct from other proteases that cannot cleave peptide bonds formed by proline residues due to its imino ring structure. POPs are highly selective as their oligopeptidase activity is restricted to the substrates of up to 30 amino acids [12]. This specificity in cleaving short peptides and exclusion of large proteins make them unique in nature. POPs are widely distributed in all the domains of life ranging from bacterial and archaeal species to humans [13]. However, unlike other serine proteases, the exact physiological role, genomic distribution and evolutionary details of POPs in bacteria is still unknown.

In most of the species, POPs are ~700 residues long with a cylindrical structure [14]. Crystal structure of bacterial POPs (bPOPs) from *Myxococcus xanthus, Sphingomonas capsulata* and *Aeromonus punctata* suggested two domain architecture with a sequentially discontinuous catalytic α/β hydrolase and a β-propeller domain [15, 16]. The α/β hydrolase domain in POPs consists of a short helical (~70 residue) N-terminal stretch and a large C-terminal region comprising of catalytic triad. The catalytic triad of Ser, Asp and His is hidden at the interface of the two structural domains. Recently, seven crystal structures of POPs of *Aeromonus punctata* suggested induced-fit mechanism of substrate entry, where addition of a substrate induces large-scale conformational changes in two domains along with alterations at the active site [16]. Studies have shown the ability of the bPOPs to cleave even 33-mer peptides [17]. POPs from different bacteria can also have differences in chain-length and sub-site specificity towards substrates [17].

POPs are one of the members of the larger ‘*POP family*’ (S9 in MEROPS), which also includes dipeptidyl peptidase IV (DPP, S9B), acylaminoacyl peptidase (ACC, S9C) and oligopeptidase B (OPB, S9A) [2, 11]. All the members of POP family are ubiquitous and exhibit restricted substrate specificities. While POP hydrolyses peptides at the carboxyl side of proline residue [12], DPP liberates dipeptides where penultimate amino acid is proline [18]. OPB cleaves at arginine and lysine bonds [19] and ACC remove N-acetylated amino acids from blocked peptides [20]. DPPs are homodimers and exist in both soluble and membrane bound forms [21–23]. POP and OPB are cytoplasmic endopeptidases, while DPP and ACC are exopeptidases. Though the sequence similarity of these four peptidases is low, they have similar three-dimensional structures with catalytic hydrolase and propeller domains. The propeller domain of POP, ACC and OPB is seven bladed, as compared to more irregular eight bladed propeller of DPP [24, 25].

POPs and POP family members are pharmaceutically important enzymes. bPOPs are considered as therapeutic agents for the oral treatment of celiac sprue, which is a small-intestinal disorder caused by abnormal response to gluten proteins [26]. High proline content of gluten makes it resistant to digestion by enzymes present in gastrointestinal tract. Treatment of gluten peptides with POPs from *Flavobacterium meningosepticum, Sphingomonas capsulatum* and *Myxococcus xanthus* has shown rapid cleavage of them [18]. Physiological role of the prokaryotic DPPs is not very clear, but there is evidence suggesting their involvement in virulence of *Porphyromonas gingivalis*, which is a major pathogen associated with adult periodontitis [27]. Similarly, OPBs are involved in the pathogenicity of *T. brucei*, the causative agent of African trypanosomiasis [9]. In trypanosome both POPs and OPBs are considered to be important virulence factor [28].

The availability of genomic information of many bacterial and archaeal species offers a great opportunity to understand the detailed distribution and biochemical role of POPs in prokaryotic lineages. Motivated by the clinical importance of POPs, we have carried out genomic identification of POPs and its homologs using exhaustive sequence search procedures. We found POPs to be widely distributed in all the classes of bacteria and archaea with diverse domain architectures. These bPOPs were depicted to be involved in protein-protein interactions and cellular signaling. Rigorous sequence searches employed in this study aided the identification of many additional POPs, which were not characterized earlier. Detailed clustering and identification of class specific sequence motifs allowed classification of bPOPs into eight subtypes. We found that multiple horizontal gene transfer events were responsible for the differential expansion of POP gene family in bacteria. To our knowledge, this is the first analysis that reports the presence of multiple POPs in many bacterial genomes.

## Results and discussion

### Genomic identification of POP homologs in bacteria and archaea

We first implemented direct and profile-based sensitive sequence search methods to identify POP homologs from 23 bacterial and 4 archaeal phyla (Additional file 1-S1a and Additional file 2). Hits were considered as ‘true’, if the sequence search algorithms could identify them with both β-propeller (POP_N) and α/β hydrolase (POP_C) domains, or with at least α/β hydrolase domain. At a stringent E-value of 10^-10^, only 1,791 POP homologs could be identified, while relaxing the E-value to 10^-3^ could capture 3,387 additional POP homologs (Additional files 3 and 4). In total, 3,010 POP homologs were collected using exhaustive Phmmer, Jackhmmer and profile-based approaches, including 2,919 bacterial and 91 archaeal POP homologs [29, 30].

The collected hits included annotated POPs, POP family members and nearby hydrolases of α/β hydrolase superfamily. Altogether, they are referred as ‘POP homologs’ in this report. In certain bacterial (*Aquificae*, *Deferribacteres*, *Elusimicrobium, Dictyoglomi, Tenricutes*) and archaeal (*Nanoarchaeota* and *Thaumarchaeota*) lineages no POP homologs could be identified. BLAST searches also failed to capture POP homologs in these phyla except in *Dictyoglomi*[31]*.* However, sequence searches against appended-PALI + database could pick at least one POP homologue in the above phyla except for *Nanoarchaeota*[32].

### Wide distribution of POP homologs in prokaryotic lineages

**Figure 1:**
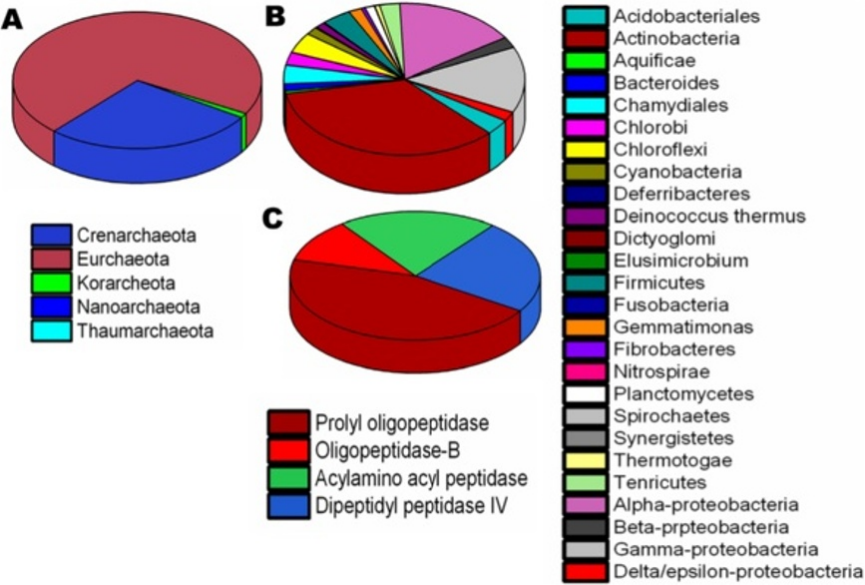
**Distribution of POP homologs in prokaryotic lineages. A)** Distribution of archaeal POP homologs. **B)** Distribution of bacterial POP homologs. **C)** Distribution of POP family members.

We noticed that all the collected POP homologs were widely distributed across all the major lineages of bacteria and archaea with apparent loss in *Nanoarchaeota*. Phylum *Actinobacteria* was identified to be the most populated with ~1000 POP homologs (Figure 1), while in archaea, many POP homologs were captured from *Euryarchaeota* and *Crenarchaeota.* In POP family, POPs were more abundant (44%) in prokaryotic lineages than DPPs (24%) and OPBs (10%) (Figure 1c Additional file 5). We could also capture all the 638 annotated POPs from prokaryotes.

### Bacterial POP homologs are both secretory and membrane proteins

**Figure 2:**
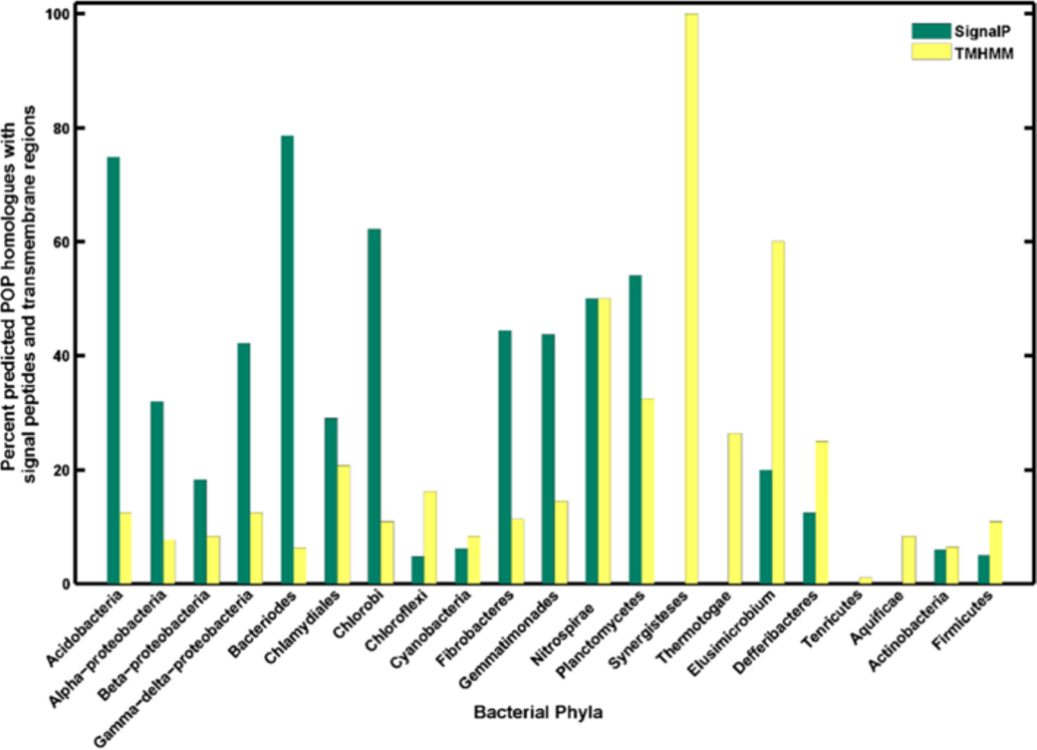
**Bacterial POP homologs with signal peptides and transmembrane regions.**

Earlier studies have shown that bPOPs are associated with the signal peptides [13]. Signal peptides are sequence motifs that permit the proteins to translocate across endoplasmic reticulum in eukaryotes and to the cytoplasmic membrane in prokaryotes. Therefore, we examined all the collected POP homologs for the presence of signal peptides. Our results showed that 20% of the POP homologs were predicted to be associated with such signals, from which 225 (35%) were annotated POPs (Figure 2). *Bacteroides* (78%) and *Acidobacteria* (75%) had maximum number of POP homologs with signal peptides, while in some bacterial phyla (e.g. *Fusobacteria, Spirochaetes, Thermotogae* and *Synergistes*) signal peptides were completely absent. POP homologs from gram-positive bacterial phyla (*Actinobacteria* and *Firmicutes*) showed relatively less number of signal peptides.

Recently, membrane-bound forms of POPs isolated from synaptosomal membranes of bovine brain were also reported [33, 34]. Cytosolic and membrane forms are different with respect to sensitivity to inhibitors, relative molecular mass, affinities for the peptide substrate and the presence of a hydrophobic membrane anchor [33, 34]. Transmembrane helix prediction by TMHMM identified 236 annotated bPOPs with single transmembrane helices located at the N-terminal [35]. Transmembrane helices were absent in POPs of phyla *Spirochaetes* and *Fusobacteria*.

### Diverse domain architectures reveal putative functions of POP homologs

**Figure 3:**
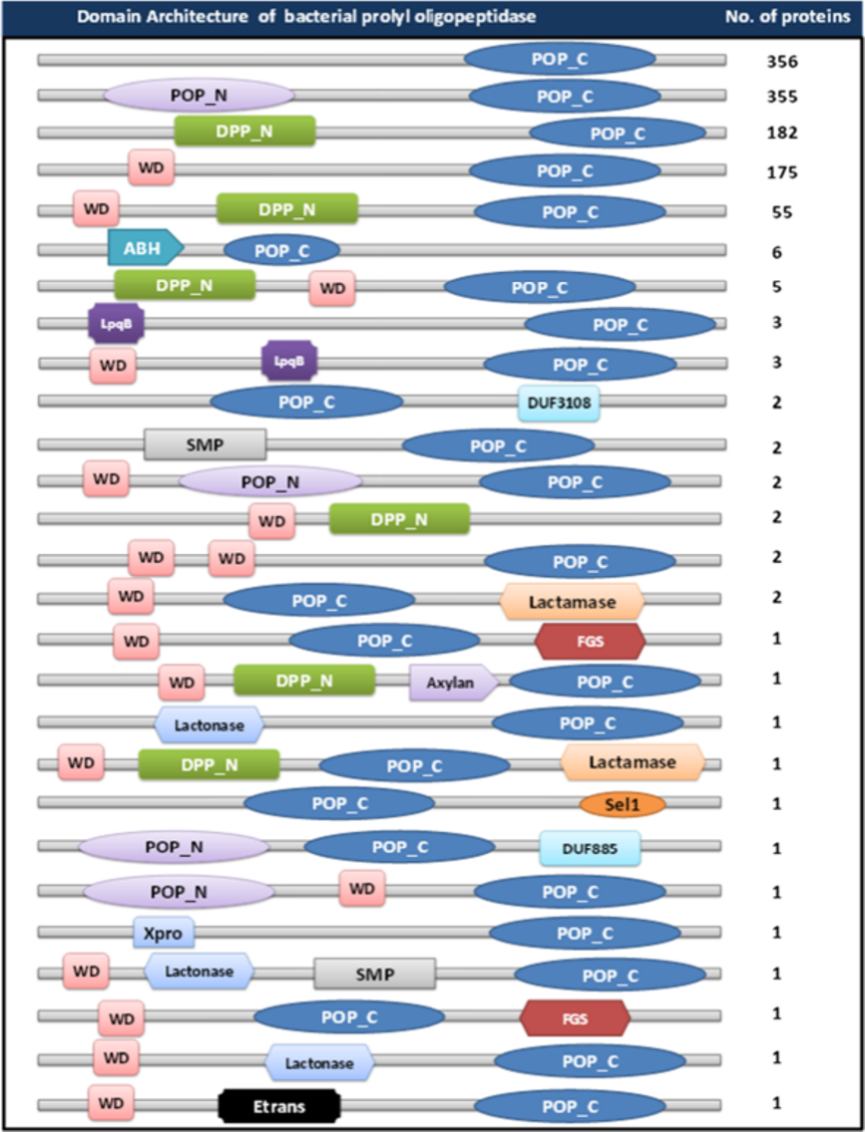
**Domain architecture of annotated bPOPs.** *Abbreviations: POP_N-prolyl oligopeptidase N-terminal, POP_C-prolyl oligopeptidase C-terminal, DPP_N-Dipeptidyl peptidase N-terminal, WD-WD domain, ABH-α/β hydrolase, LpqB –Lipoprotein,DUF- Domain of unidentified function, SMP-SMP-30/gluconolactonase/LRE-like region, FGS-Formylglycine-generating sulfatase, Axylan-Acetyl xylan, Xpro -X-Pro dipeptidyl-peptidase, Etrans-Eukaryotic translation.*

**Figure 4:**
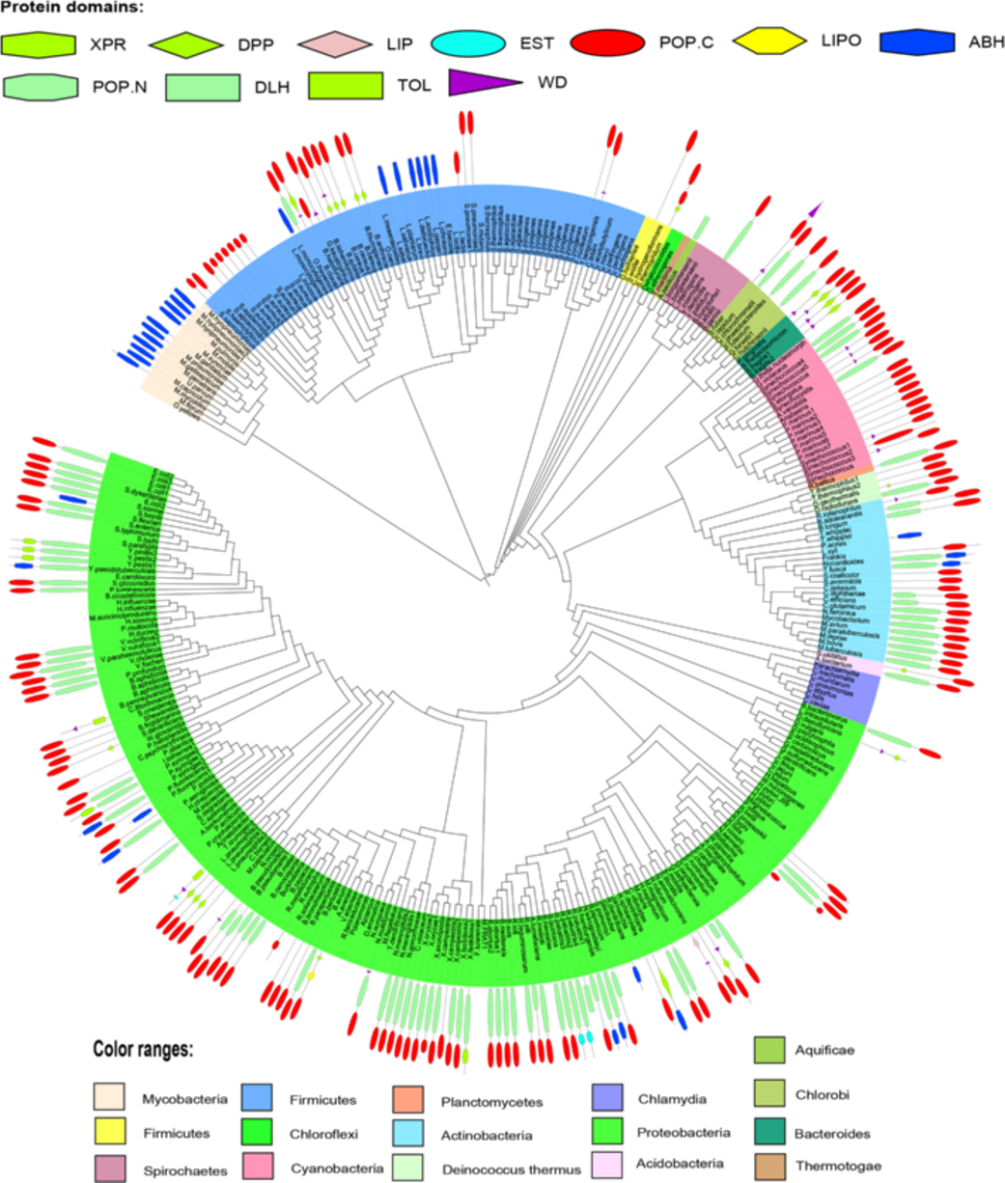
**Domain architecture of POP homologs mapped on the species tree of bacteria.** *Abbreviations: DPP-Dipeptidyl peptidase, LIP-Lipoprotein LpqB, EST-esterase, POP_N-prolyl oligopeptidase N-terminal domain, POP_C-prolyl oligopeptidase C-terminal domain, LIPO-lysophospholipase, ABH-α/β hydrolase, XPR -X-Pro dipeptidyl-peptidase, DLH-Dienelactone hydrolase, TOL- TolB amino-terminal, WD-WD domain.*

**Figure 5:**
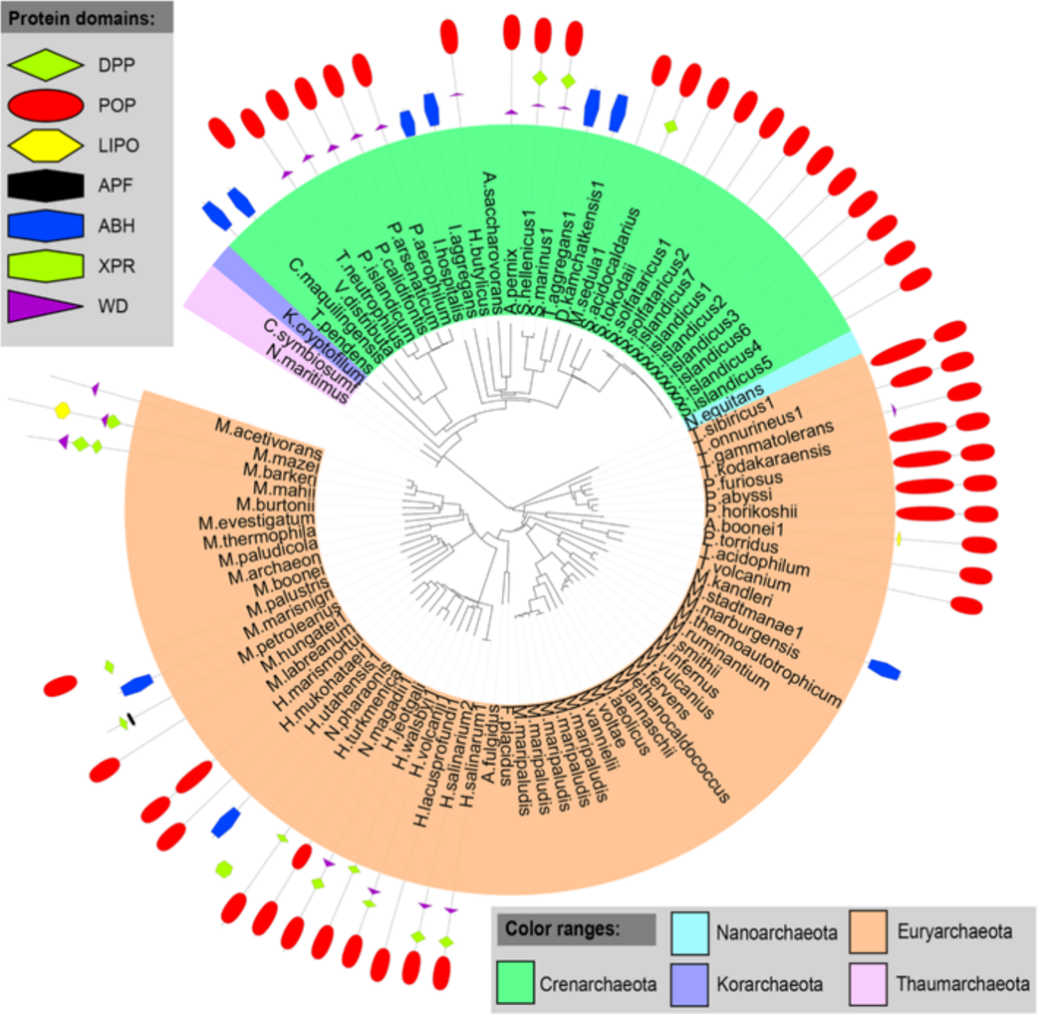
**Domain architecture of POP homologs mapped on the species tree of archaea.** *Abbreviations: DPP-Dipeptidyl peptidase, POP_N-prolyl oligopeptidase N-terminal domain, POP_C-prolyl oligopeptidase C-terminal domain, LIPO-lysophospholipase, APF- Anaphase-promoting complex, ABH-α/β hydrolase, XPR-X-Prodipeptidyl-peptidase, WD-WD domain.*

We then investigated the coexisting domains to understand the possible biological functions of POP homologs in the prokaryotic lineages. Bacterial and archaeal POP homologs were associated with 105 and 8 different domain architectures respectively (Figure 3, Additional file 6). Both the archaeal and bacterial POP homologs share similar domain architectures suggesting similar function of POP homologs in these two kingdoms. Domain architectures of POP homologs were also mapped on species tree of bacteria and archaea. As shown in Figure 4, POPs were associated with diverse domain combinations in *Proteobacteria,* while in mycobacterial species POPs were replaced by other hydrolases. Within a phylum, anomalous distribution of POPs was observed. Mapping of domain architecture on archaeal species tree depicted presence of only C-terminal POP domain in most of the organisms, while full-length POP domains were observed in a few species of *Crenarchaeota* (Figure 5).

POP homologs were frequently associated with protein-protein interaction domains *e.g.* PDZ and tetratricopeptide (TPR) repeats. Two of the ‘C-terminal processing peptidases’ (S41) had PDZ domains, which are associated with signaling proteins of bacteria, plants and higher order organisms. PDZ domains are involved in assembly of large protein complexes, thereby coordinating and guiding the flow of regulatory information [36, 37]. PDZ domains present in peptidase S41 of *Candidatus Solibacter usitatus* (YP_821861) and *Roseiflexus* (YP_001276641) were associated with WD40 and DPP domains. TPR repeat motifs facilitate interaction with other proteins. These motifs were also related with hydrolase domain in *Candidatus Solibacter usitatus* (YP_824720). TPR-proteins are also associated with multi-protein complexes, and are involved in functioning of chaperones, cell-cycle, transcription and protein transport complexes [38, 39].

POP homologs were also associated with signaling modules such as WD-repeats. Proteins with WD-repeat exhibit high degree of functional diversity [40–42]. Some archaeal POPs were also predicted to be associated with WD-repeats suggesting conserved function of POPs in the two domains of life. Besides WD repeats, POP homologs were also related with Sel1 repeats, which are subfamily of TPR sequences. In prokaryotes, these repeats allow proteins to be membrane attached and mediate interaction between bacterial and eukaryotic host cells [43, 44]. One of the POP proteins from *Ferrimonas balearica* (YP_003914375) was predicted to be associated with Sel1 repeats.

Bacterial POP homologs were also found to co-exist with several DNA-binding modules of transcription regulatory domains. Numerous bacterial transcription regulatory proteins bind DNA *via* a helix-turn-helix motif [45]. These are sequentially diverse transcriptional activators and most of them are known to negatively regulate their own expression. Transcription regulatory domain is associated with response regulator receiver domain and plays an important role in DNA-binding and regulation of transcription [46, 47]. POP homologs that co-existed with bacterial regulatory domains include *Candidatus Solibacter usitatus* (YP_827731) and *Caulobacter segnis* (YP_003594106), and those with transcription regulatory domain include four homologs (two each from *Actinobacteria* and *Gammaproteobacteria* (YP_888147 (*Mycobacterium smegmatis*), YP_001759306 (*Shewanella woodyi*), YP_735011 (*Shewanella sp. MR-4*) and YP_954217 (*Mycobacterium vanbaalenii*)). Targeted deletions of the predicted accessory domains will be beneficial to understand the related biological functions.

### Different cellular localization of annotated bPOPs

We have also examined the cellular localization of annotated bPOPs to infer the possible functions of POP in more detail. Prediction of cellular localization using PSORT-b also revealed cytoplasmic nature of the annotated POPs (176 versus 115 POPs which were predicted to be periplasmic) (Additional files 4 and 7) [48]. Interestingly, we predicted some of these POPs to be localized in cell wall, cytoplasm and outer membranes of bacteria and archaea. Different bacterial phyla depicted differences in preferred cellular localization of POPs. For example, in phylum *Proteobacteria*, most of the POPs were periplasmic in nature. Clustering analysis of the predicted cytoplasmic and periplasmic POPs resulted in a clear separation of cytoplasmic and periplasmic POPs with a few exceptions.

### Phylogenetic analysis of annotated bPOPs shows high co-clustering

**Figure 6:**
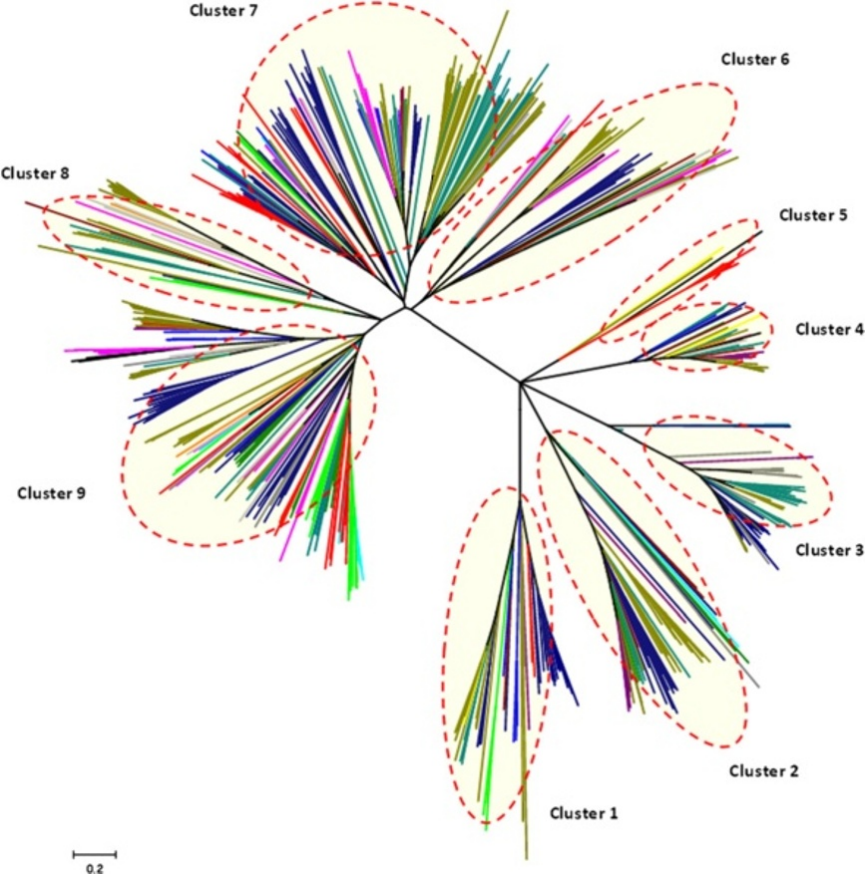
**Phylogenetic analysis of annotated bPOPs.** Color code: *Thermotogae*-cyan, *Firmicutes*-lime, *Chloroflexi*-green, *Deinococcus-thermus*-blue, *Chlorobi*-magenta, *Actinobacteria*-blue, *Acidobacteria*-yellow, *Alphaproteobacteria*-teal, *Betaproteobacteria*-grey, *Gammaproteobacteria*-olive, *Deltaproteobacteria*-blue, *Bacteriodetes*-black, *Planctomycetes*-black, *Cyanobacteria*-purple, *Gemmatimonadetes*-Red branch with species name in black, *Spirochaetes*-pink branch with species name in black, *Fibrobacteres*-light grey, *Archaebacteria*-red. Nine distinct clusters are marked in red.

To investigate the differences in the annotated bPOPs, we next performed phylogenetic clustering of 638 annotated POPs that showed nine distinct clusters, with co-clustering among members of different phyla (Figure 6). This co-clustering trend and absence of phylum-specific clusters suggested high conservation of POPs within bacterial lineages. Genus *Shewanella* of marine metal-reducing bacteria was highly populated with considerable number of annotated bPOPs in all the nine clusters. Similarly, archaeal POPs were also co-clustered well with other bPOPs. This co-clustering suggested the possibility of lateral transfer of POP genes among bacteria and between archaeal and bacterial species (Additional files 4 and 8).

### Unique sequence signature motifs depict diverse sequence properties

**Figure 7:**
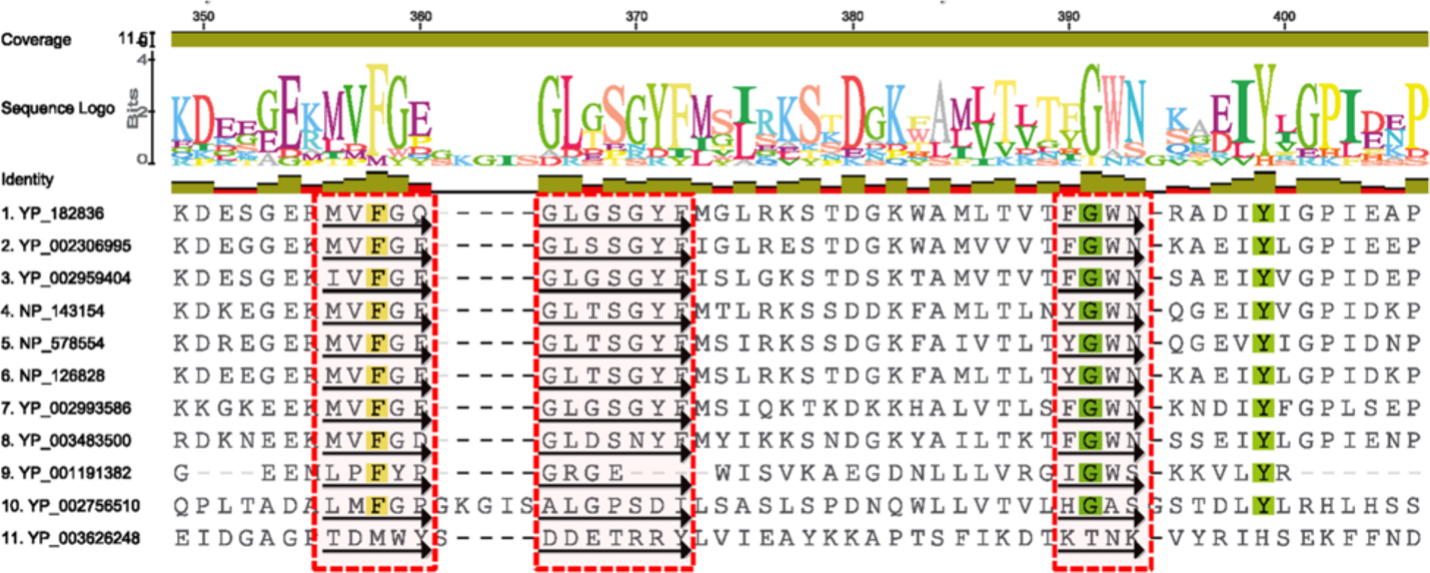
**Part of sequence alignment of POPs of the fifth cluster representing class specific motifs.** Red boxes and arrows represent class specific motifs, only 90% conserved residues are colored.

To further analyse the co-clustering trend of annotated bPOPs, we identified conserved class specific sequence motifs. An alignment stretch was considered as a ‘conserved motif’, if 95% of the sequences had conserved amino acids at least at three consecutive positions. From these highly conserved sequence motifs, we next identified class specific motifs. A ‘class-specific motif’ was defined as a sequence motif in a cluster, which was completely absent from all the other clusters. In the first and seventh clusters (Figure 6), no class-specific motifs were observed. Figure 7 shows a part of the alignment of fifth cluster of bPOPs representing class specific motifs. Detailed analysis of motifs of all the clusters was carried out to understand their relative position on the structure of bPOPs. Class-specific motifs of second, sixth, eighth and ninth cluster were localized in the hydrolase domain, while motifs of cluster third, fourth and fifth were distributed on both the domains (see Additional files 9 and 10 for details).

### Classification of annotated bPOPs into eight subtypes

**Figure 8:**
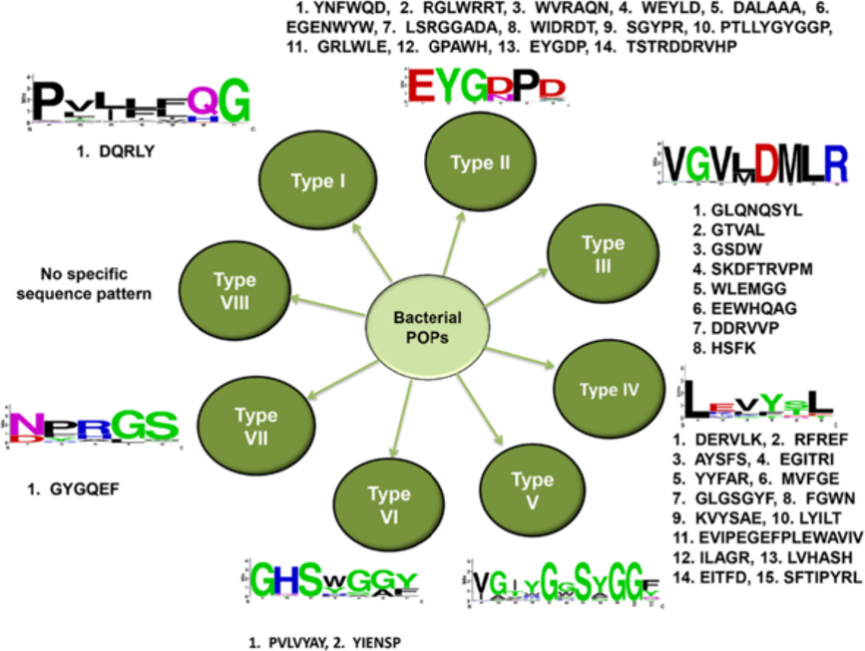
**Different subtypes of bPOPs and the motifs associated with them.** Most frequently observed motif is shown in WebLogo. Details of these motifs are present in Additional files 4, 9 and 10.

Detailed analysis of class specific sequence motifs indicated high sequence variations in annotated bPOPs. Therefore, on the basis of identified class specific motifs, we propose a classification of bPOPs into eight different subtypes as shown in Figure 8. Some of these class-specific motifs were surface exposed, depicting their possible involvement in protein-protein interactions with other interacting partners (for details see Additional file 4), while some other motifs were located in the core of protein, near functionally important residues, which could possibly cause differences in interaction with the versatile substrates of POPs.

### Subtypes of bPOPs differ in the conservation of functionally important residues

We then investigated the conservation of functionally important residues in different subtypes of bPOPs. Detailed analysis revealed high conservation of catalytic triad residues in all the subtypes. However, high number of non-permissible amino acid replacements was observed to be concentrated at two sites―Ser-571 and Thr-573 (numbering according to the bPOP crystal structure, PDB id: 2BKL), which are located at the interface of two domains. These sites were replaced by non-polar and positively charged amino acids in most of the subtypes of bPOPs (Additional file 11). These two residues were also situated in vicinity of Arg-572 and Ile-575 that were reported to be crucial for the incoming peptide substrate in bPOPs. W-575, which is important for the substrate binding was conserved in some of the bPOPs, while in a few other bPOPs it was substituted by other amino acids. Altogether, the hydrophobic environment required for the substrate binding was not conserved in all the bPOPs. These findings strengthen our hypothesis that the proposed bPOP subtypes can also be different with respect to the possible substrate. Mutation experiments of these functionally important residues can provide further insights about their role in the catalytic activity and substrate specificity.

### Divergence of POP family members

Besides analysing the co-clustering pattern of annotated bPOPs, we have also examined the divergence of POP family members. From all the 3,010 collected POP homologs, we could obtain 1,421 POP family members including 638 annotated POPs, 156 OPBs, 293 ACCs and 334 DPPs. These members were used to construct a joint phylogenetic tree, where a set of bacterial carboxylesterases (20 sequences) were considered as an outgroup. We observed that OPBs and DPPs were distinctly clustered, while the ACCs and POPs were dispersed all over the tree (Additional files 12 and 13). bPOP family tree was contradictory to the tree earlier reported by Venäläinen *et al.,* where distinct clusters of POP family members from all the domains of life were observed [13].

The phylogenetic analysis also suggested high divergence of other POP family members. Some of the POPs have diverged from the rest of the POP family members before OPBs, followed by the divergence of ACCs and DPPs. ACCs were diverged along with some of the other POPs, since distinct cluster of ACCs could not be obtained. This clustering pattern was confirmed by generating additional phylogenetic tree, where only DPPs, OPBs and ACCs were considered to understand their phyletic distribution (Additional file 4). If POPs were excluded from the phylogenetic tree, other members of POP family formed distinct clusters, which revealed that POPs were responsible for the observed co-clustering among the POP family members.

### Anomalous distribution of annotated bPOPs revealed many multi-POP bacterial genomes

**Figure 9:**
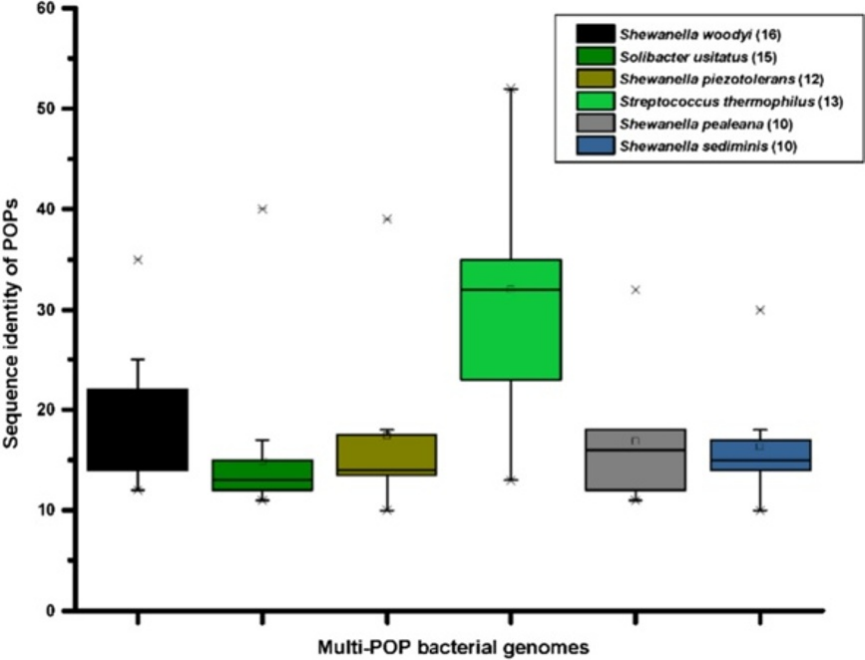
**Sequence variations of the POPs of multi-POP bacterial genomes.** Numbers of putative POPs identified in these genomes are shown in brackets.

While performing the sequence analysis, we noticed high variations in the number of annotated POP genes in bacterial genomes, ranging from no POPs to multiple copies of POPs within a genome. Overall, out of 269 identified bacterial genomes with annotated POPs, 148 had a single copy of POP gene. The overrepresentation of POP was particularly observed in genus *Shewanella* of *Gammaproteobacteria*, where most of the species had multiple copies of POP gene. One of the interesting examples of multi-POP proteome was *Shewanella woodyi* with 16 POPs sharing an average sequence identity of 15% (ranging from 8 to 35%). Moreover, we could identify 12 copies of POP gene in *Shewanella piezotolerans,* and 10 copies each in *Shewanella pealeana* and *Shewanella sediminis.* Besides genus *Shewanella,* 15 POP genes were also identified in *Solibacter usitatus.* High sequence variations in paralogs of POP suggested that they are not closely related to each other, except in *S. thermophilus* genome (Figure 9, Additional file 4). These multiple POPs within a genome also differ in their cellular localizations (Additional file 1-S1a).

### Horizontal gene transfer as a driving force for the expansion of POP gene family in bacteria

Examination of the complete genomes of bacterial and archaeal lineages showed considerable variations in the number of annotated POP genes within a genome. Horizontal gene transfer (HGT) and gene duplication are the two driving forces, which may lead to expansion of gene families in prokaryotic systems [49]. We have studied the expansion of POP gene family in more detail using POP rich genus *Shewanella*. Members of genus *Shewanella* have been described from diverse habitats, including deep cold-water marine environments to shallow Antarctic ocean habitats, to hydrothermal vents and freshwater lakes [50]. We examined sequence similarity and chromosomal positioning to determine if HGT is prevalent in these genomes. Chromosomal mapping of 16 annotated POP genes of *S. woodyi* depicted non-co-localization, representing possible HGT events during evolution (Additional file 14). Only two genes (6118839 and 6118846 bearing a low sequence identity of 20%) were found to be slightly closer on the genome, still separated by six other genes. Similar patterns were also observed in POP genes of other species of *Shewanella.* This suggests possibility of multiple HGT events during the evolution of these bacteria (Additional file 1-S1b and 4).

### Annotation of uncharacterized POP homologs of bacterial lineages

During sensitive sequence searches, we could identify many hypothetical proteins with POP-like signatures. We have implemented various approaches such as protein domain identification, secondary structure prediction, protein fold prediction and GO annotation mapping to characterize 38 hypothetical sequences as POPs and 159 proteins as α/β hydrolases [29, 51–53]. A hypothetical sequence was annotated as POP if an annotated POP query picked the sequence at least at an E-value of 10^-3^ and it had similar domain architecture (with both α/β hydrolase and β-propeller domains). During this analysis some partial POPs comprising of only catalytic domain were also identified (Additional file 1, S1c). RPS-BLAST (Reversed Position Specific BLAST) using four different profiles (annotated POPs, ACCs, DPPs and other hydrolases of α/β hydrolase superfamily) was carried out to further scan each unannotated sequence, thereby confirming that these sequences are α/β hydrolase superfamily members [31].

### Limitations of the computational methods used in this study

Although we have used multiple methods for the detailed analysis of POP homologs from the bacterial and archaeal lineages, yet the current study has certain limitations. Instead of relying on any one-sequence search method, here, we have employed multiple sequence search algorithms to detect all possible homologs. We validated all the obtained hits by mapping functional domains and active site residues, yet the possibility of obtaining false positives cannot be ignored.

The complete absence of POP genes from some of the bacterial phyla could be because of the caveats of the sequence search algorithms. It is possible that POPs of such phyla are so diverged that most of the methods failed to identify them, including current remote homology detection methods. Therefore, further experimental characterization of these genomes is essential to conclude the presence or absence of POPs. The available computational methods to predict protein domains, cellular localizations and signal peptides of protein sequences are also associated with wrong predictions.

During this study, we have also encountered many incomplete POP sequences with either missing N-terminal hydrolase domain (e.g. YP_001519174.1, *Acaryochloris marina*) or with incomplete propeller domain (e.g. YP_003320038.1, *Sphaerobacter thermophilus*) or with only hydrolase domain (e.g. Mycoplasma genomes). These partial POPs could be due to errors in the available gene prediction algorithms. Additionally, wrong or incomplete annotation of collected protein sequences could also lead to another source of error. Experimental validation of these reported sequences would help in improving the current annotations of the corresponding genomes.

## Conclusion

In this study, we have performed an exhaustive computational analysis of POPs in prokaryotic lineages. Our analysis revealed wide distribution, high diversity and functional importance of POPs in the analysed bacterial and archaeal genomes. Many novel domain combinations were identified in bPOPs, emphasizing the need for systematic studies to understand them further. In addition, we noticed different selection pressures on propeller and hydrolase domains.

The POP family has primarily expanded by multiple HGT events. POP paralogs differ considerably with respect to sequence, cellular localization and domain architectures, suggesting functional divergence and maintenance by natural selection [54, 55]. Finally, the proposed classification of bPOPs will help in understanding the sequence specific characteristics and structural differences of these POPs.

In conclusion, our systematic analysis of POPs in bacterial and archaeal species will aid in a better understanding of these proteins. This bacterial POP repertoire will also facilitate comparative and functional genomics studies, and experimental characterization of unique domain combinations. With the rapidly growing numbers of sequenced genomes, our work can be considered as a benchmark in the extension of such analysis to other less-studied protein families.

## Methods

### Classification of bacterial proteomes

1,202 prokaryotic (1,112 bacterial, 90 archaeal) proteomes were downloaded from NCBI. These proteomes were further classified according to NCBI Taxonomy database into 23 bacterial and 4 archaeal phyla. These bacterial species include diverse groups of extremophiles, pathogens, model organisms and symbiotic bacteria.

### Search for POP homologs in bacterial genomes

All the prokaryotic proteomes were scanned for the presence of POPs using BLAST and HMM-SEARCH (Additional file 15) [29, 31]. Instead of relying on a single method, multi-search procedures were employed to identify POP homologs from the bacterial proteomes. Sequence searches were performed at two different E-values to identify POP homologs from all the targeted proteomes. These methods include:

- Direct approach: A direct approach was implemented by extracting the annotated POP sequences from the proteomes. If a proteome was found to have an annotated POP, it was considered as a query sequence to obtain more POP homologs. For identification of POP homologs Phmmer and Jackhmmer (iterative searching as PSI-BLAST) of the HMMER suite were used at stringent E-values of 10^-3^ and 10^-10^[29]. Homologs were collected according to phyla and redundant sequences from each phylum were removed with CD-HIT [56]. This approach helped in identification of POP homologs where an annotated POP could already be identified.
- Profile based approach: Bacterial and archaeal specific profiles were generated with a member (POP) from each phylum using HMMbuild. This could help in picking nearest POP homologs from all the collected bacterial proteomes. Besides archaeal and bacterial-specific profiles, an integrated profile was also generated using bacterial, archaeal and eukaryotic POPs. Furthermore, HMMsearch was performed using these three different profiles at two different E-values of 10^-3^ and 10^-10^. This approach helped in picking homologs from proteomes, where annotated POPs were absent.

### Enrichment of true homologs

Homologs collected at the relaxed E-value of 10^-3^ were further confirmed as ‘true homologs’ by constructing the database of sequences obtained at stringent E-value of 10^-10^. All the sequences found at E-value of 10^-3^ were considered as query and BLAST was performed against the database of ‘true homologs’. Domain definitions were identified using HMMScan against Pfam database for all the collected hits [57]. A hit was considered as a ‘true’ hit if it had both POP and α/β hydrolase domains.

To be sure that none of the POPs were missed, BLASTP was also performed against some of the selected proteomes. Furthermore, some phyla were also screened using an indirect approach of appending bacterial proteomes to PALI + database, which comprises of trusted homologs of proteins of known three-dimensional structures [31].

### Relative density of distribution of POP homologs in bacterial genomes

Relative abundance of POP homologs was calculated using relative density. Relative density is defined as the total number of serine proteases (POP homologs) identified in a taxonomic lineage by the total number of genomes of that lineage, which were considered for the study. Relative occurrence is defined as the total number of POP homologs identified in a taxonomic lineage by the total number of gene products in a taxonomic lineage.

### Assignment of co-existing domains, transmembrane regions and signal peptides

Domain assignments were mapped using HMMPfam (E-value of 10^-3^), which scans the sequences against HMMs of Pfam database. Transmembrane regions of all the hits were examined using TMHMM [36], which is a highly accurate HMM based method to predict transmembrane regions. Since POP is found to have protein-sorting signals, all the collected sequences were searched for the signal peptides using SignalP [58]. SignalP is the most accurate program based on neural networks to clearly distinguish signal peptides from the transmembrane regions [59]. Secondary structure prediction and fold assignments of unannotated proteins were carried out using PSIPRED and GenTHREADER, respectively [51, 52].

### Multiple sequence alignment and phylogenetic analysis

Multiple sequence alignment was carried out using MUSCLE [60]. The multiple sequence alignment was further utilized for performing phylogenetic analysis of collected POP homologs using MEGA5 [61]. In this analysis, we have employed neighbor-joining method, where clusters with bootstrap value greater than 50% were considered for the detailed analysis. Multiple sequence alignment was represented using WebLogo and Geneious [62]. iTOL (Interactive Tree Of Life) was used for mapping domain architecture on the species tree of bacteria and archaea [63]. MODELLER was employed for homology modeling of POP sequence, where bPOP (PDB id: 2BKL) was chosen as a template [64]. Modeled POP structures were further used for mapping sequence motifs. Functionally important residues were scored using Scorecons, where a score of more than 0.7 was considered to be significant [65].

## Electronic supplementary material

Additional file 1: Table S1a: Sequenced bacterial and archaeal genomes analysed in this study. Table S1b: BLAST searches of *Shewanella* genomes, cellular localization and domain architecture of multiple POP proteins of *Shewanella*. Table S1c Annotated hypothetical proteins. (XLS 214 KB)

Additional file 2: **Distribution of the sequenced genomes of bacterial and archaeal lineages.** Distribution of sequenced bacterial **(A)** and archaeal **(B)** genomes. (PDF 2 MB)

Additional file 3: **Schematic representation of the pipeline followed to collect true homologs.** (PDF 2 MB)

Additional file 4: **A. Enrichment of true homologs. B**. Relative abundance and occurrence. **C**. Other domain architectures of POP homologs. **D**. Different cellular localization. **E**. Cluster-wise sequence identity. **F**. Structural mapping of sequence motifs of each cluster. **G**. Functional domains of annotated bacterial POPs are conserved and glycine rich. **H**. Divergence of POP family members. **I**. Detailed analysis of POPs of *Shewanella*. **J**. Sequence similarity searches to understand HGT events. (DOCX 2 MB)

Additional file 5: **Distribution of POP-family members in different bacterial and archaeal phyla.** (PDF 76 KB)

Additional file 6: **Domain architecture of POP homologs.** *Abbreviations: POP_C-prolyl oligopeptidase C-terminal, DPP_N-Dipeptidyl peptidase N-terminal, WD-WD domain, ABH-α/β hydrolase, DLH-Dienelactone hydrolase, EstPHB-Esterase PHB, Xpro -X-Pro dipeptidyl-peptidase, ABC- ABC transporter, TFB- transcription regulatory domain, TAP- TAP-like protein, TPR- Tetratrico peptide repeats, CNB- cyclicnucleotide binding, Osmc- OsmC-like protein, ASST- Arylsulfo transferase, KAS- beta-ketoacyl synthase, AT- Acyl transferase, AD- Alcohol dehydrogenase, ZnD- Zinc binding dehydrogenase, Branched chain aa- Branched chain amino acid, PRT- Phosphoribosyl transferase, DUF- Domain of unidentified function, BRP- Bacterial regulatory protein, FGS- Formylglycine generating sulfatase, S-layer- S-layer homology, TRD- Transcriptional regulatory domain, SMP-SMP-30/gluconolactonase/LRE-like region.* (PDF 432 KB)

Additional file 7: **Cellular localization of annotated bPOPs.** (PDF 3 MB)

Additional file 8: **Detailed phylogeny of annotated bPOPs.** Color code: *Thermotogae*-cyan, *Firmicutes*-lime, *Chloroflexi*-green, *Deinococcus-thermus*-blue, *Chlorobi*-magenta, *Actinobacteria*-blue, *Acidobacteria*-yellow, *Alphaproteobacteria*-teal, *Betaproteobacteria*-grey, *Gammaproteobacteria*-olive, *Deltaproteobacteria*-blue, *Bacteriodetes*-black, *Planctomycetes*-black, *Cyanobacteria*-purple, *Gemmatimonadetes*-Red branch with species name in black, *Spirochaetes*-pink branch with species name in black, *Fibrobacteres*-light grey, *Archaebacteria*-red. (PDF 62 KB)

Additional file 9: **Sequence alignment and class specific motifs of each cluster.** An arrow represents class specific motifs. (PDF 20 MB)

Additional file 10: **Cluster-wise mapping of sequence motifs on the structure of POPs.** (PDF 920 KB)

Additional file 11: **A) Mapping of non-permissible amino acid replacement sites on bPOP structure.** Color code: Amino acid replacement sites-red, functionally important residues-cyan and green, active site-magenta, catalytic domain-yellow and propeller domain-blue. **B)** Cluster-wise conservation and replacement of functionally important residues. Top row shows functionally important residues as reported in mammalian POPs. Active site residues are represented in pink. Non-permissible amino acid replacements are shown in yellow. Numbers represent percentage conservation in different clusters. (PDF 345 KB)

Additional file 12: **Phylogenetic analysis of POP family members.** Color code: POP-blue, DPP-red, ACC-magenta, OPB-green, carboxyesterase-brown. (PDF 2 MB)

Additional file 13: **Detailed phylogeny of POP family members.** Color code: POP-blue, DPP-red, ACC-magenta, OPB-green, carboxyesterase-brown. (PDF 89 KB)

Additional file 14: **Chromosomal mapping of 16 POP genes of** ***Shewanella woodyi.*** Color code: Purple and green color represents GC content and GC skew in this genome. (PDF 173 KB)

Additional file 15: **Schematic of sequence searches followed in this work.** (PDF 684 KB)
