# Supplementary material for: Distribution, classification, domain architectures and evolution of prolyl oligopeptidases in prokaryotic lineages
Source: BMC Genomics. 2014 Nov 18;15(1):985. doi: 10.1186/1471-2164-15-985 (PMC4522959; doi:10.1186/1471-2164-15-985)
Supplement: Supplementary file 2 — Additional file 2: Distribution of the sequenced genomes of bacterial and archaeal lineages. Distribution of sequenced bacterial (A) and archaeal (B) genomes. (PDF 2 MB) [file 12864_2014_7072_MOESM2_ESM.pdf]

A

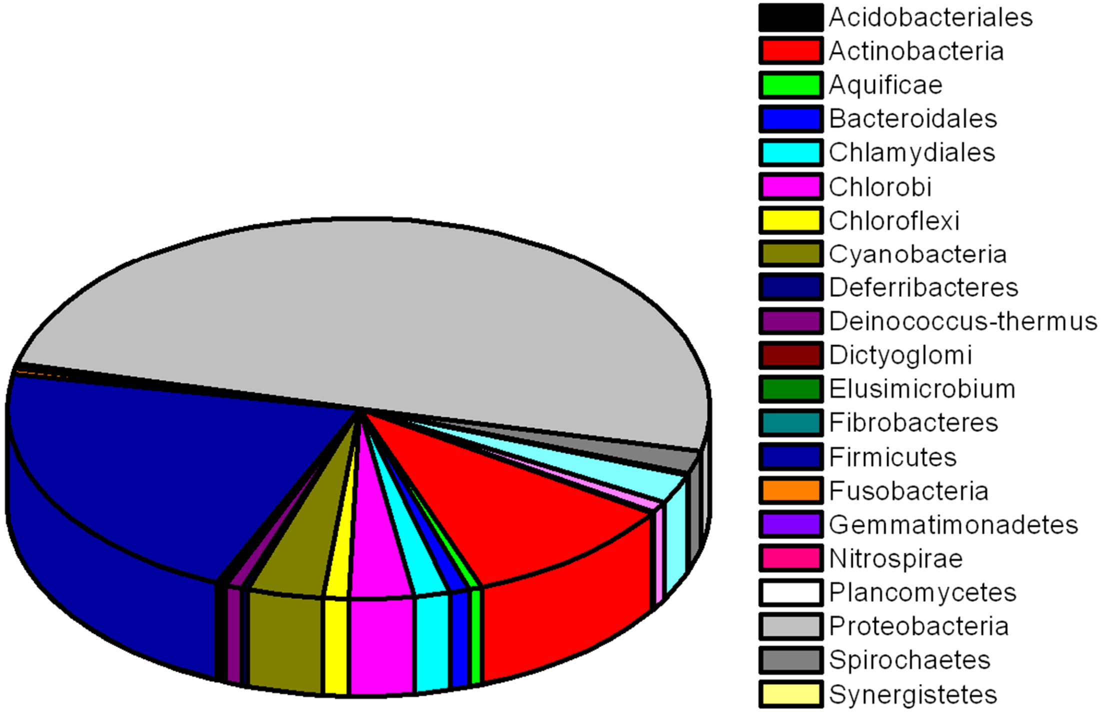
Acidobacteriales
Actinobacteria
Aquificae
Bacteroidales
Chlamydiales
Chlorobi
Chloroflexi
Cyanobacteria
Deferribacteres
Deinococcus-thermus
Dictyoglomi
Elusimicrobium
Fibrobacteres
Firmicutes
Fusobacteria
Gemmatimonadetes
Nitrospirae
Plancomycetes
Proteobacteria
Spirochaetes
Synergistetes
Tenricutes
Thermotogae

B

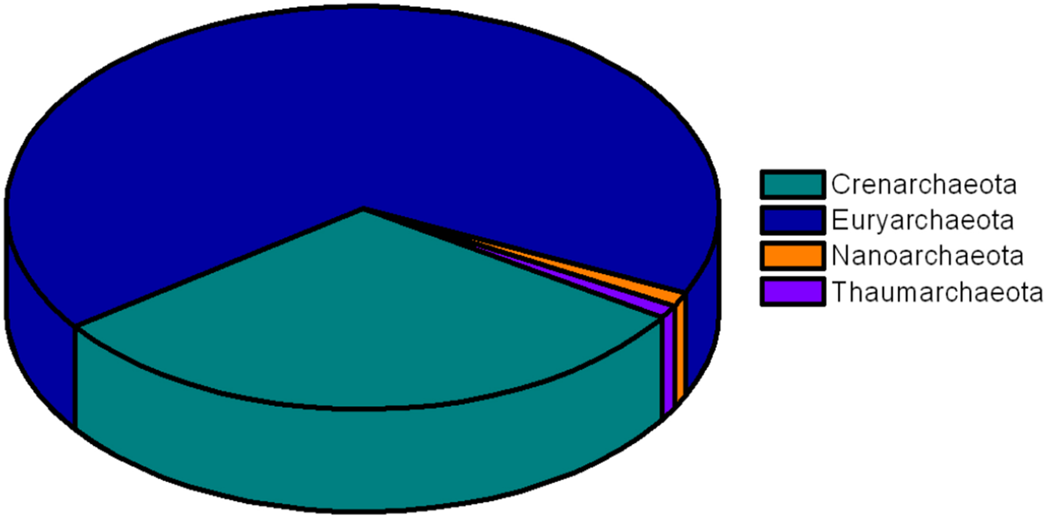
Crenarchaeota
Euryarchaeota
Nanoarchaeota
Thaumarchaeota
