# Supplementary figures and images for: Distribution, classification, domain architectures and evolution of prolyl oligopeptidases in prokaryotic lineages
Source: BMC Genomics. 2014 Nov 18;15(1):985. doi: 10.1186/1471-2164-15-985 (PMC4522959; doi:10.1186/1471-2164-15-985)

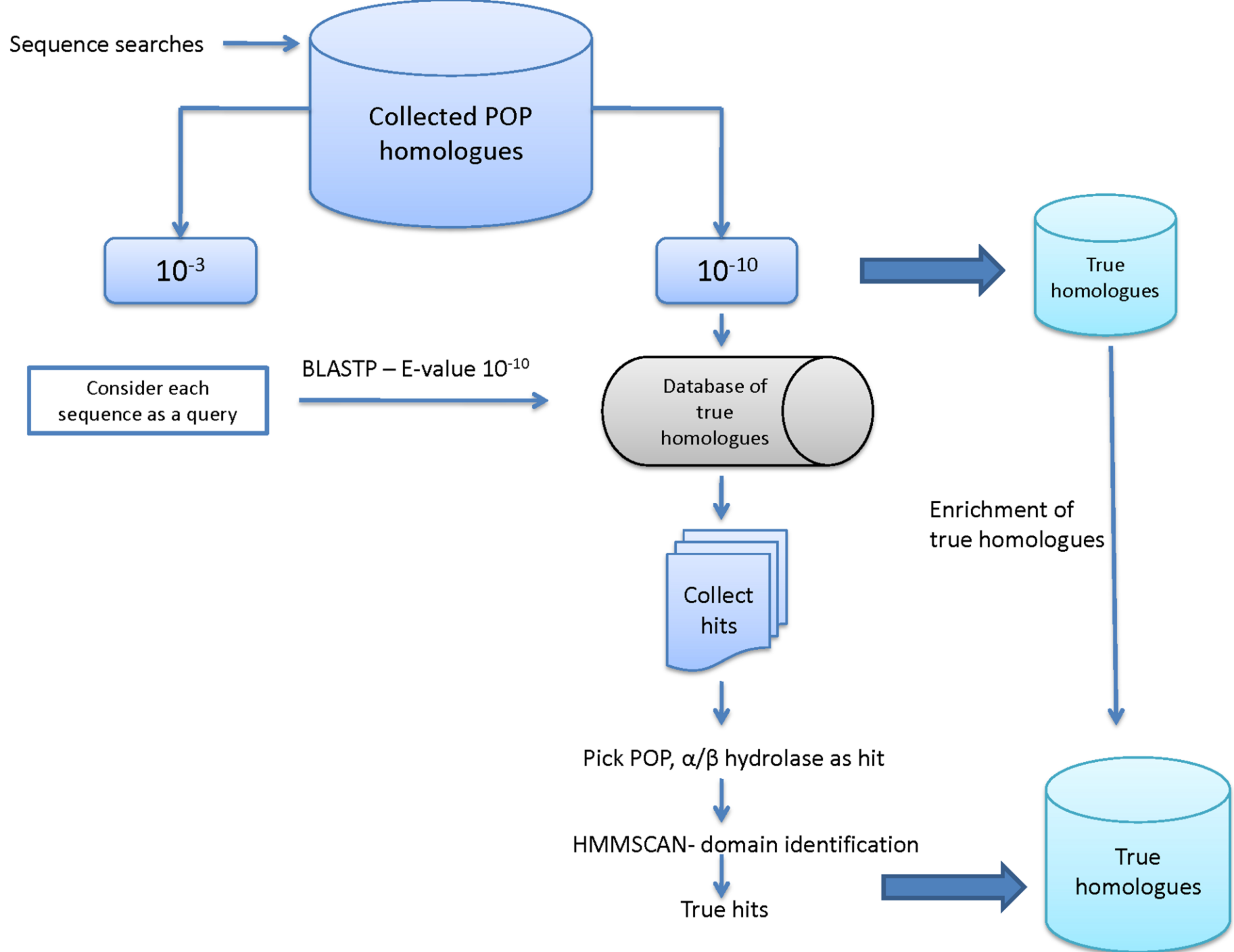

Supplement: Supplementary file 3 — Additional file 3: Schematic representation of the pipeline followed to collect true homologs. (PDF 2 MB) [file 12864_2014_7072_MOESM3_ESM.pdf]

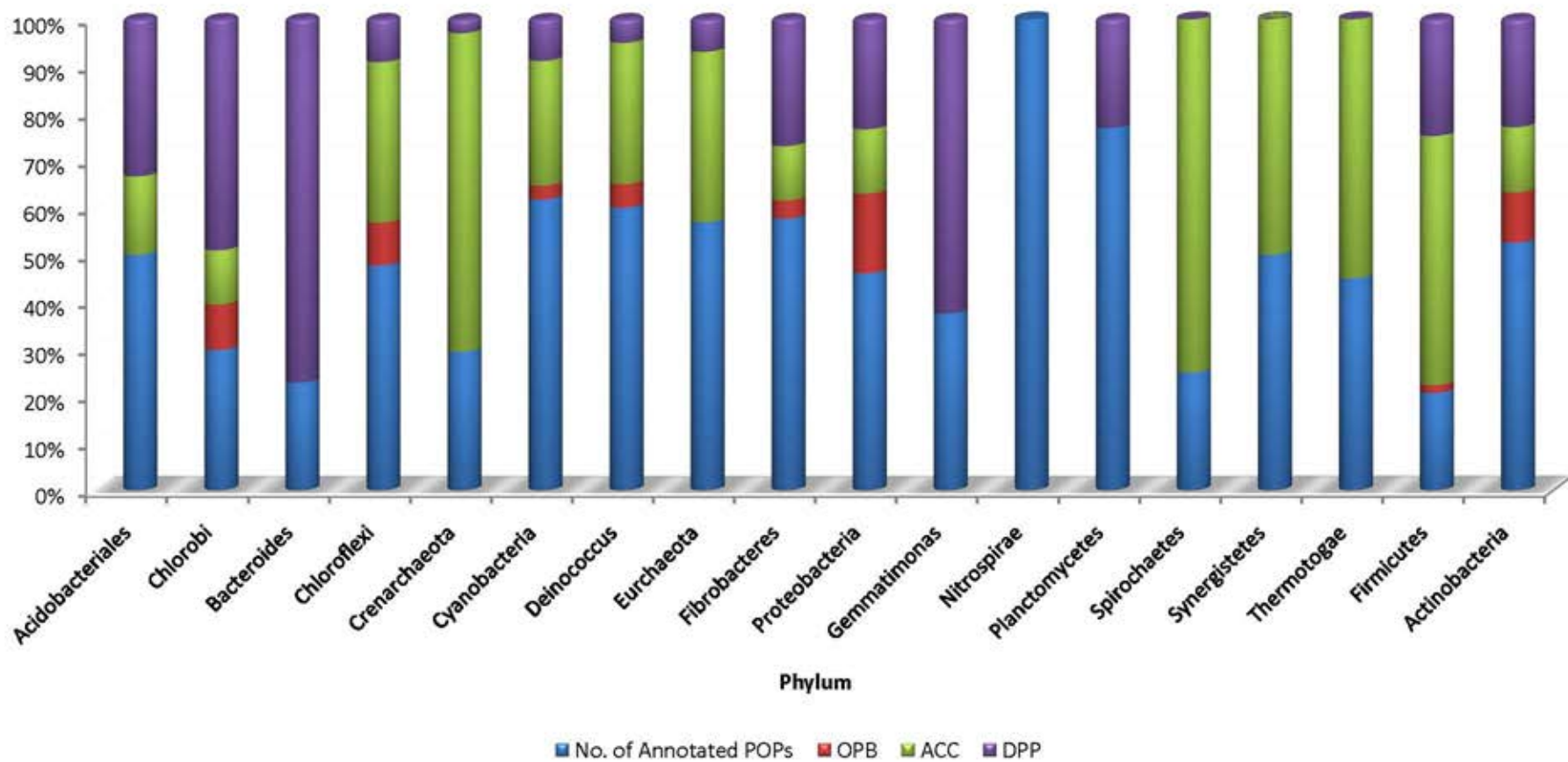

Supplement: Supplementary file 5 — Additional file 5: Distribution of POP-family members in different bacterial and archaeal phyla. (PDF 76 KB) [file 12864_2014_7072_MOESM5_ESM.pdf]

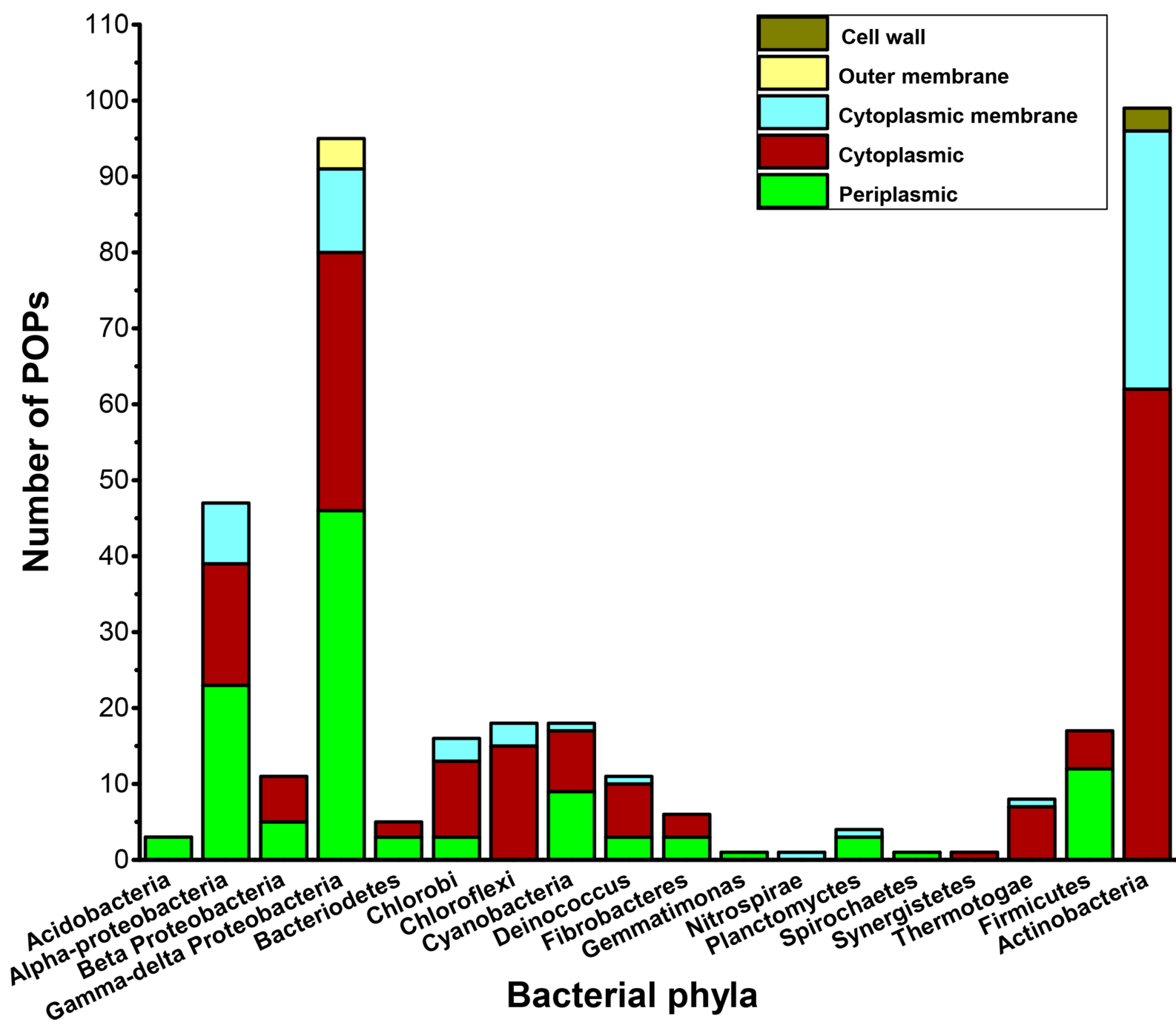

Supplement: Supplementary file 7 — Additional file 7: Cellular localization of annotated bPOPs. (PDF 3 MB) [file 12864_2014_7072_MOESM7_ESM.pdf]

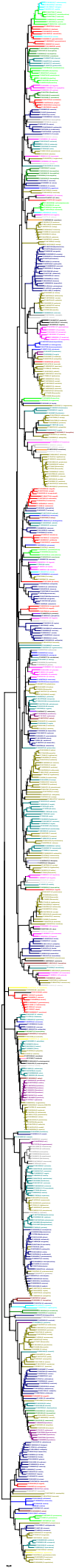

Supplement: Supplementary file 8 — Additional file 8: Detailed phylogeny of annotated bPOPs. Color code: Thermotogae-cyan, Firmicutes-lime, Chloroflexi-green, Deinococcus-thermus-blue, Chlorobi-magenta, Actinobacteria-blue, Acidobacteria-yellow, Alphaproteobacteria-teal, Betaproteobacteria-grey, Gammaproteobacteria-olive, Deltaproteobacteria-blue, Bacteriodetes-black, Planctomycetes-black, Cyanobacteria-purple, Gemmatimonadetes-Red branch with species name in black, Spirochaetes-pink branch with species name in black, Fibrobacteres-light grey, Archaebacteria-red. (PDF 62 KB) [file 12864_2014_7072_MOESM8_ESM.pdf]

## CLUSTER-2

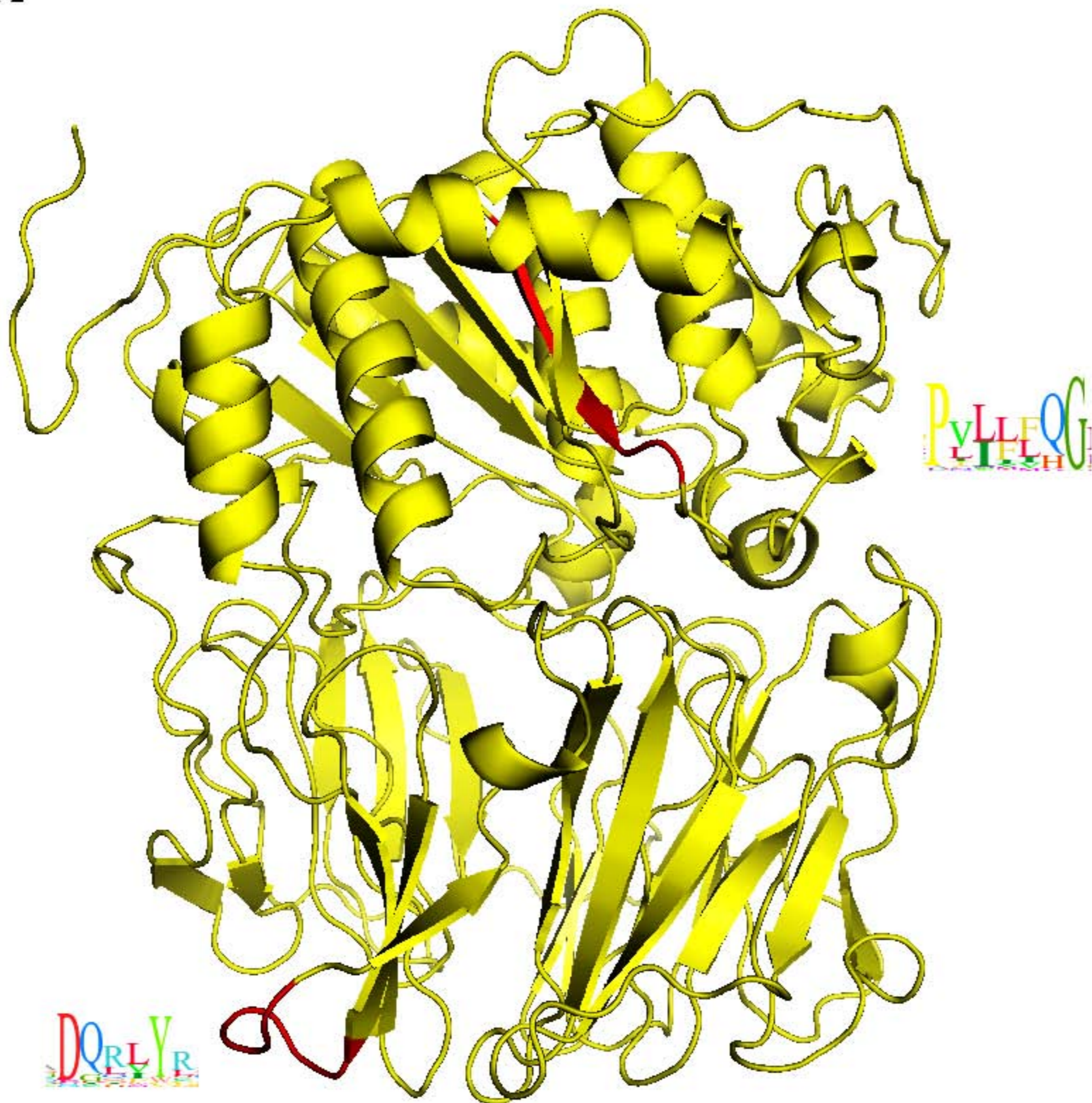

CLUSTER-3

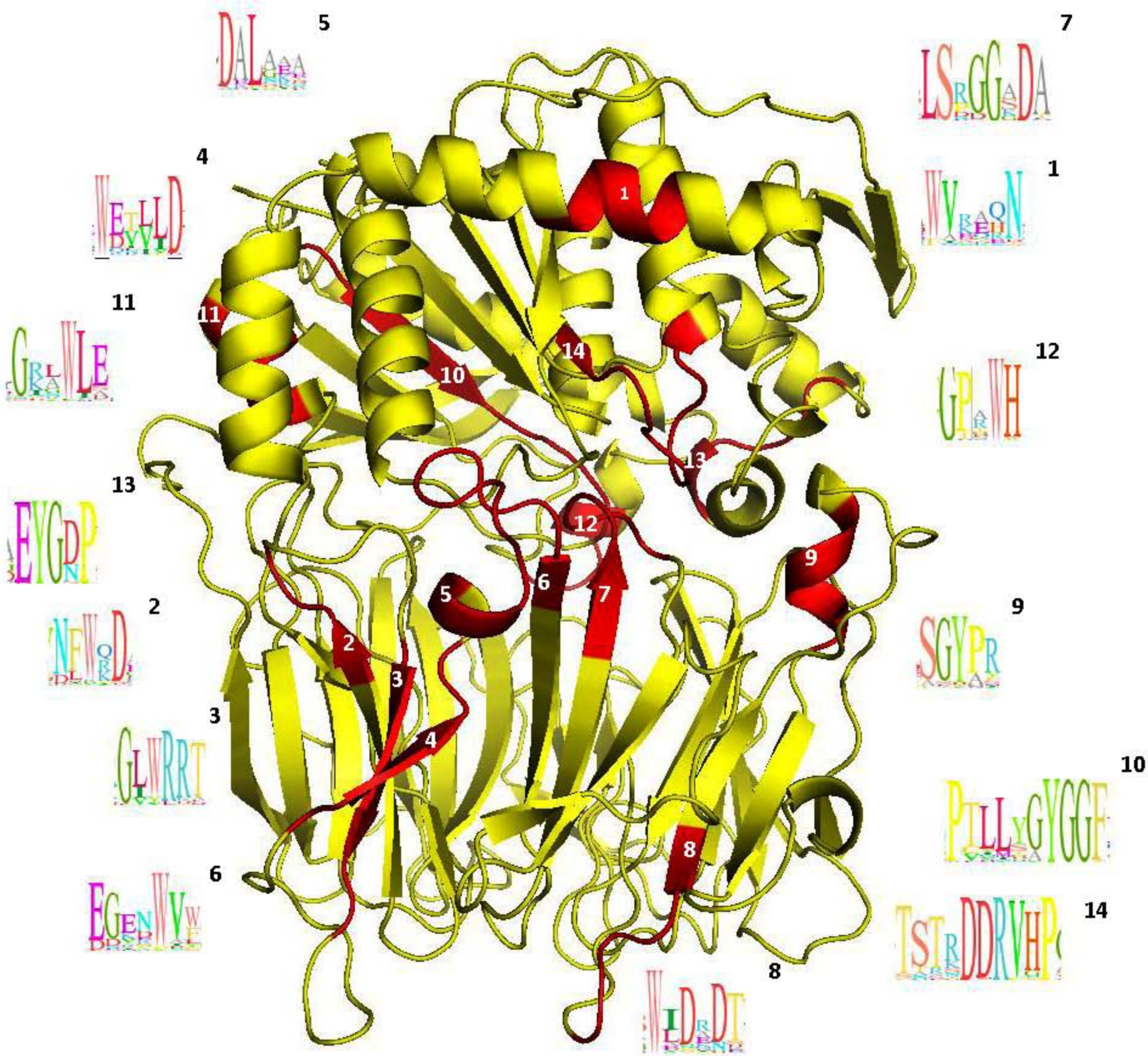

# CLUSTER-4

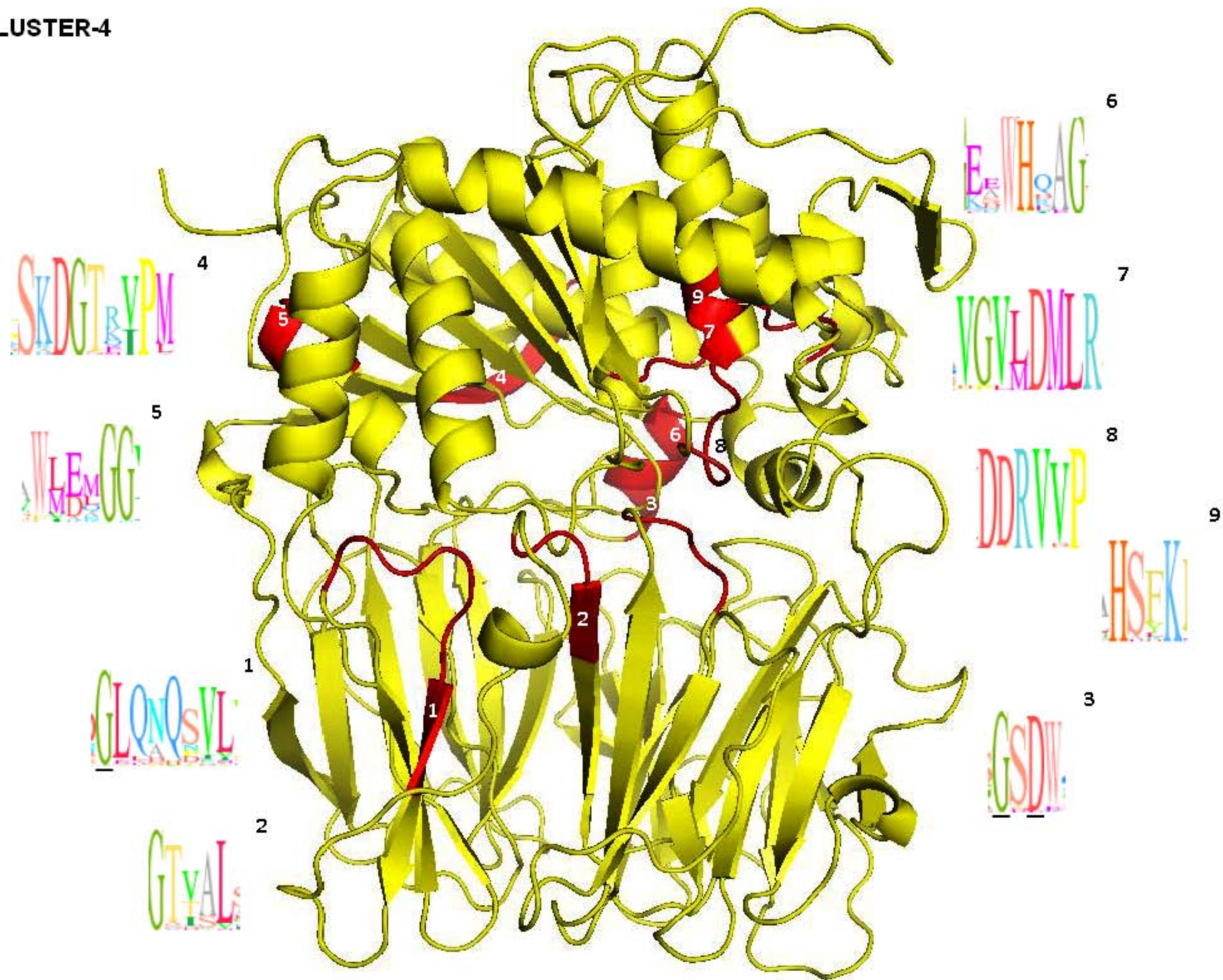

# CLUSTER-5

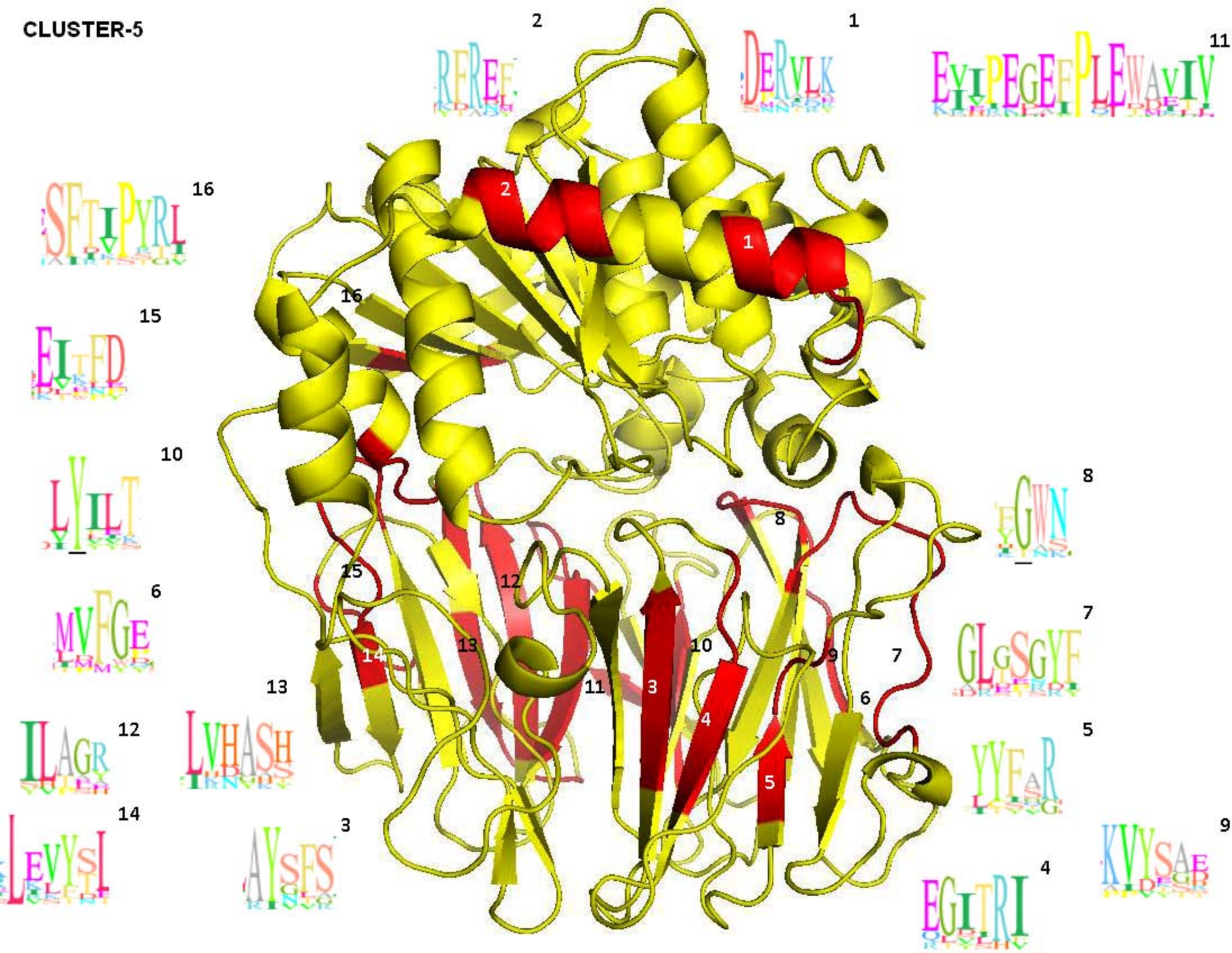

## CLUSTER-6

VGIYGGSYGG

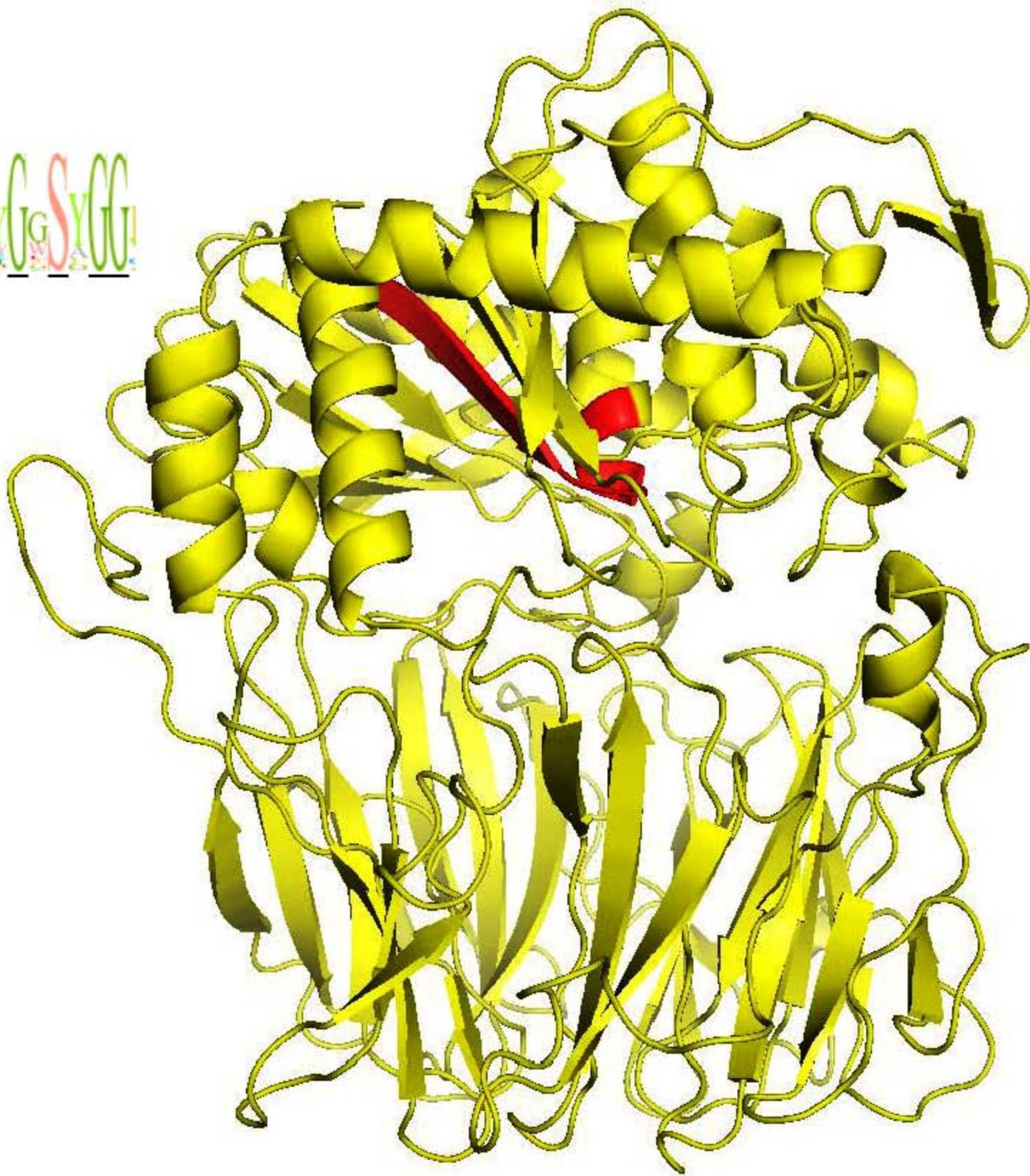

CLUSTER-8

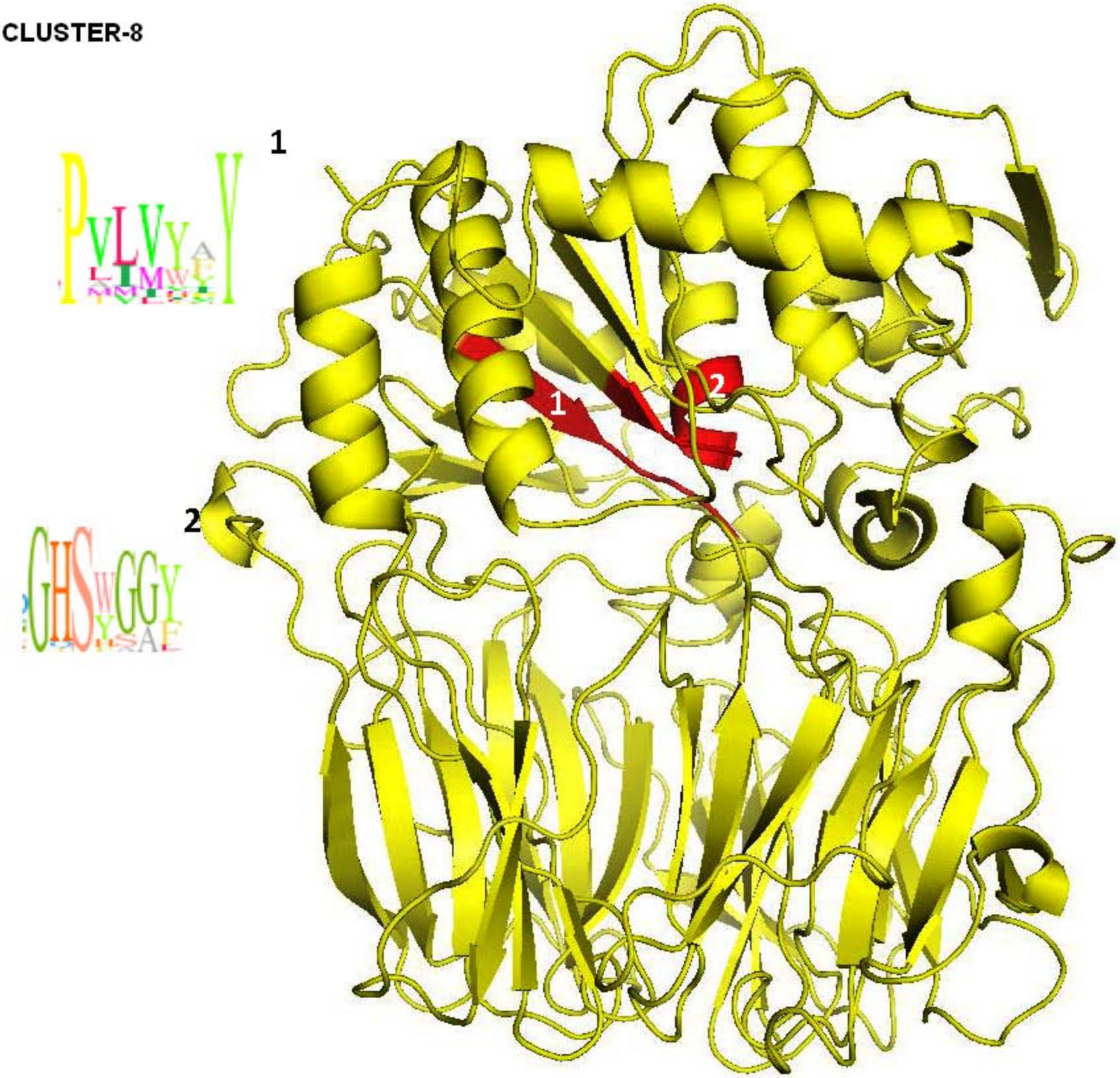

CLUSTER-9

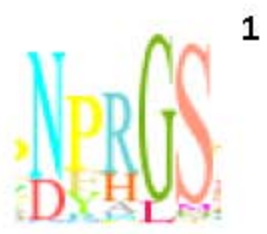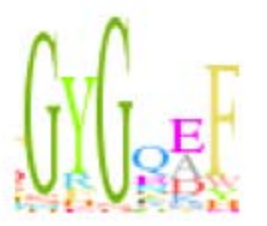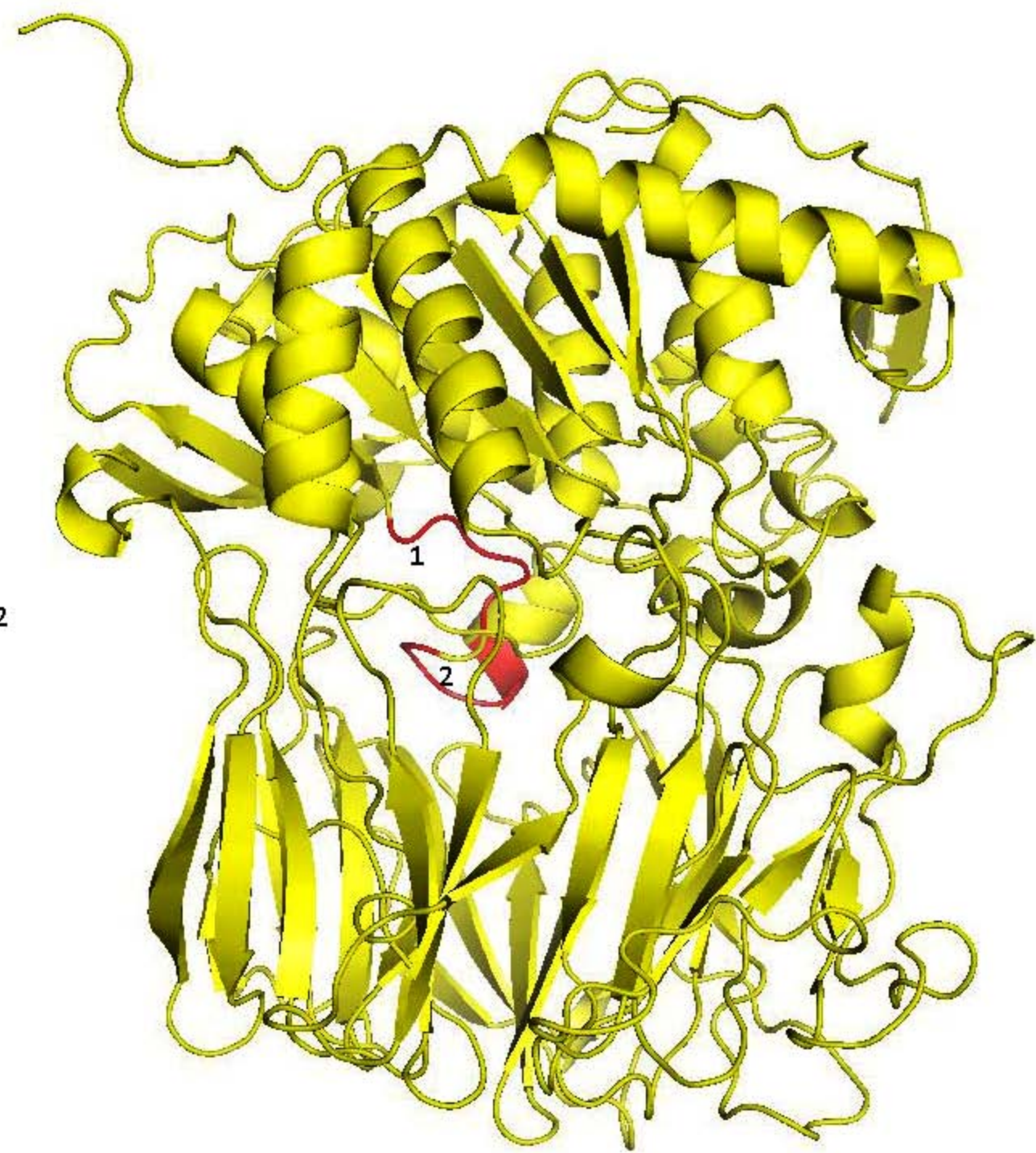

Supplement: Supplementary file 10 — Additional file 10: Cluster-wise mapping of sequence motifs on the structure of POPs. (PDF 920 KB) [file 12864_2014_7072_MOESM10_ESM.pdf]

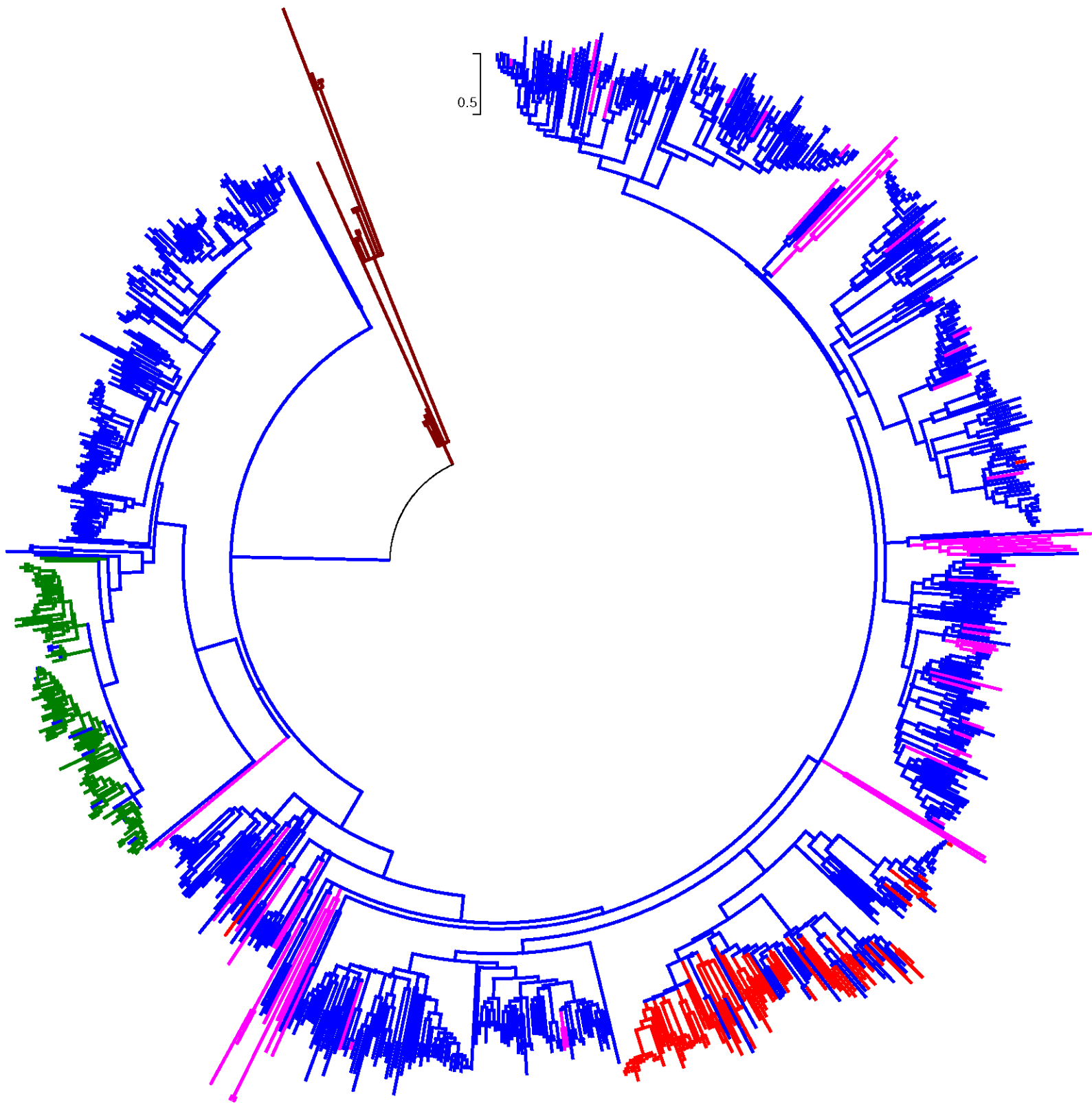

Supplement: Supplementary file 12 — Additional file 12: Phylogenetic analysis of POP family members. Color code: POP-blue, DPP-red, ACC-magenta, OPB-green, carboxyesterase-brown. (PDF 2 MB) [file 12864_2014_7072_MOESM12_ESM.pdf]

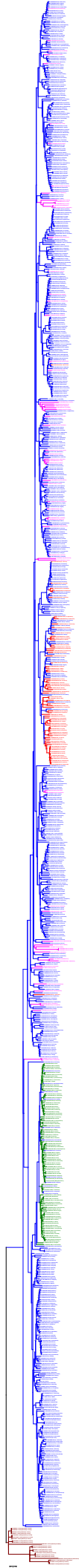

Supplement: Supplementary file 13 — Additional file 13: Detailed phylogeny of POP family members. Color code: POP-blue, DPP-red, ACC-magenta, OPB-green, carboxyesterase-brown. (PDF 89 KB) [file 12864_2014_7072_MOESM13_ESM.pdf]

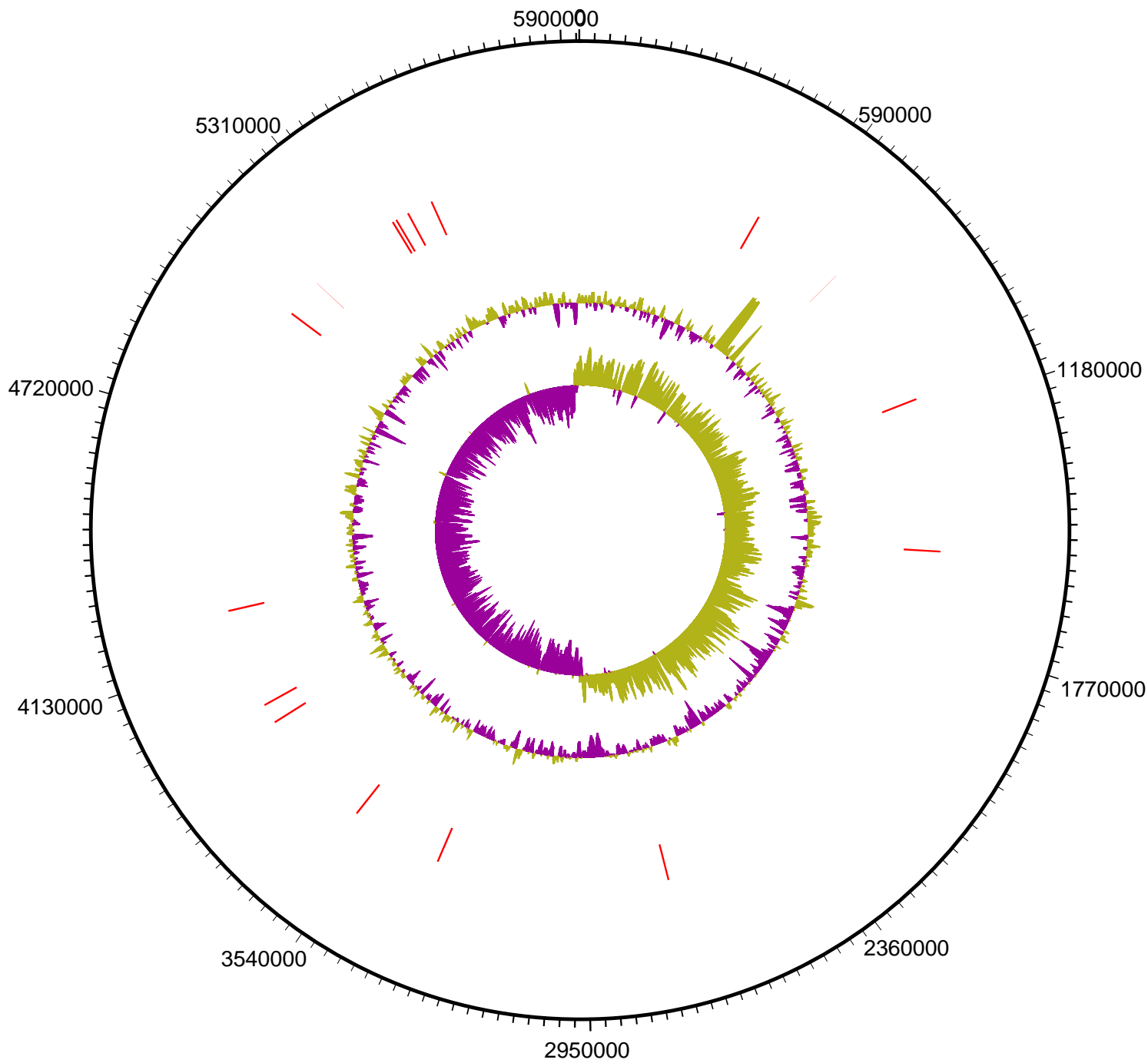

Supplement: Supplementary file 14 — Additional file 14: Chromosomal mapping of 16 POP genes of Shewanella woodyi. Color code: Purple and green color represents GC content and GC skew in this genome. (PDF 173 KB) [file 12864_2014_7072_MOESM14_ESM.pdf]

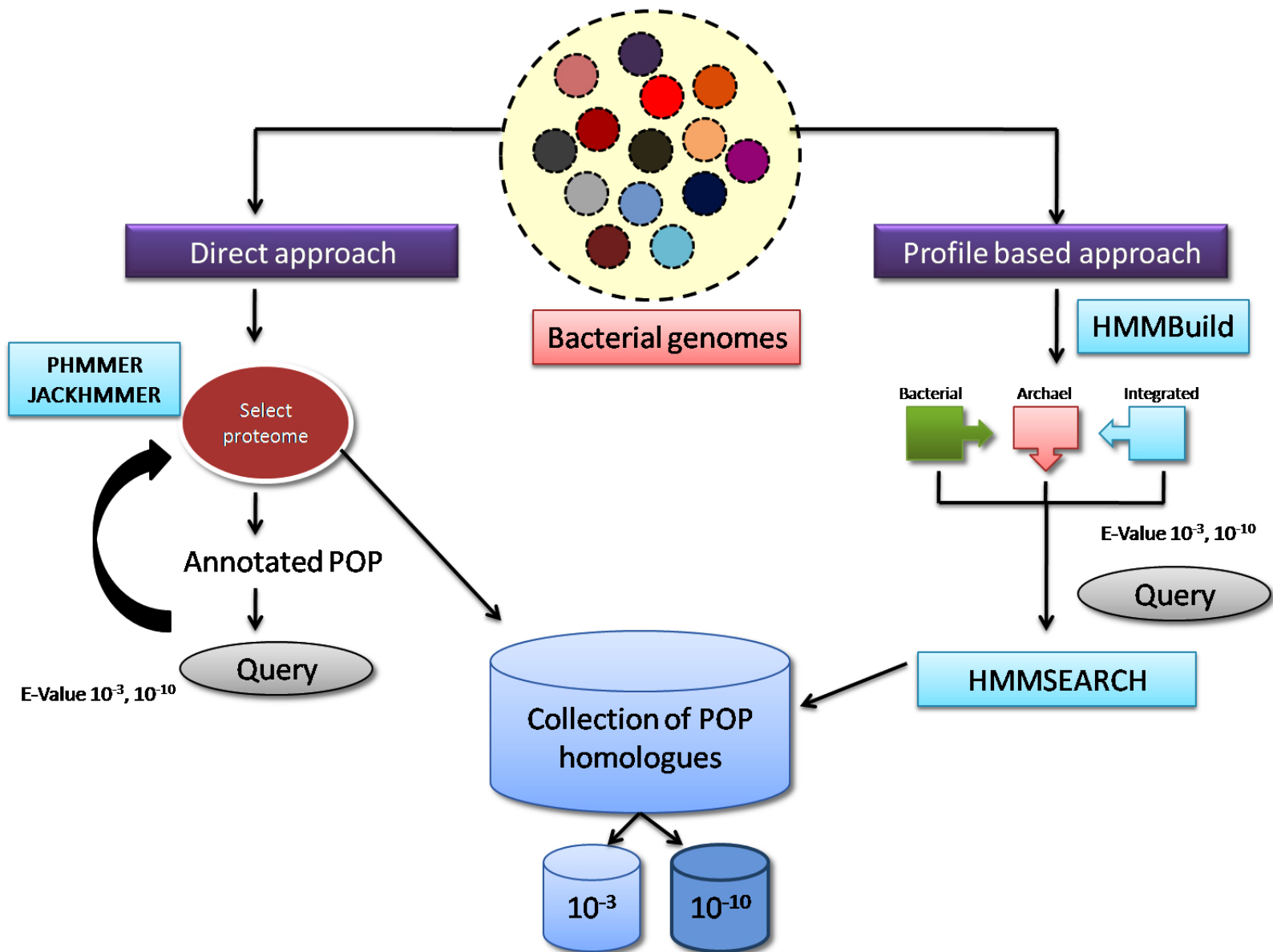

Supplement: Supplementary file 15 — Additional file 15: Schematic of sequence searches followed in this work. (PDF 684 KB) [file 12864_2014_7072_MOESM15_ESM.pdf]
