# Supplementary material for: Distribution, classification, domain architectures and evolution of prolyl oligopeptidases in prokaryotic lineages
Source: BMC Genomics. 2014 Nov 18;15(1):985. doi: 10.1186/1471-2164-15-985 (PMC4522959; doi:10.1186/1471-2164-15-985)
Supplement: Supplementary file 4 — Additional file 4: A. Enrichment of true homologs. B. Relative abundance and occurrence. C. Other domain architectures of POP homologs. D. Different cellular localization. E. Cluster-wise sequence identity. F. Structural mapping of sequence motifs of each cluster. G. Functional domains of annotated bacterial POPs are conserved and glycine rich. H. Divergence of POP family members. I. Detailed analysis of POPs of Shewanella. J. Sequence similarity searches to understand HGT events. (DOCX 2 MB) [file 12864_2014_7072_MOESM4_ESM.docx]

**A. Enrichment of true homologues**

Enrichment of true homologues was carried out using a database of hits collected at the stringent E-value of 10^-10^ as shown in Additional file 3. POP homologues collected at the relaxed E-value of 10^-3^ were considered as a query sequence to perform BLASTP against this database which led to the identification of 1219 additional POP homologues.

**B. Relative abundance and occurrence**

Relative abundance or relative density was determined as a ratio of number of homologues of POP identified in a phylum by the total number of targeted bacterial proteomes. Relative density of *Actinobacteria* and *Planctomycetes* was observed to be highest, suggesting higher number of POP homologues per proteome in these classes of bacteria. This high density of POP homologues infers the importance of POPs in these bacteria. However, archaea showed lower representation of POP homologues. Similarly, relative occurrence indicated that POP homologues were highest in *Elusimicrobium*.


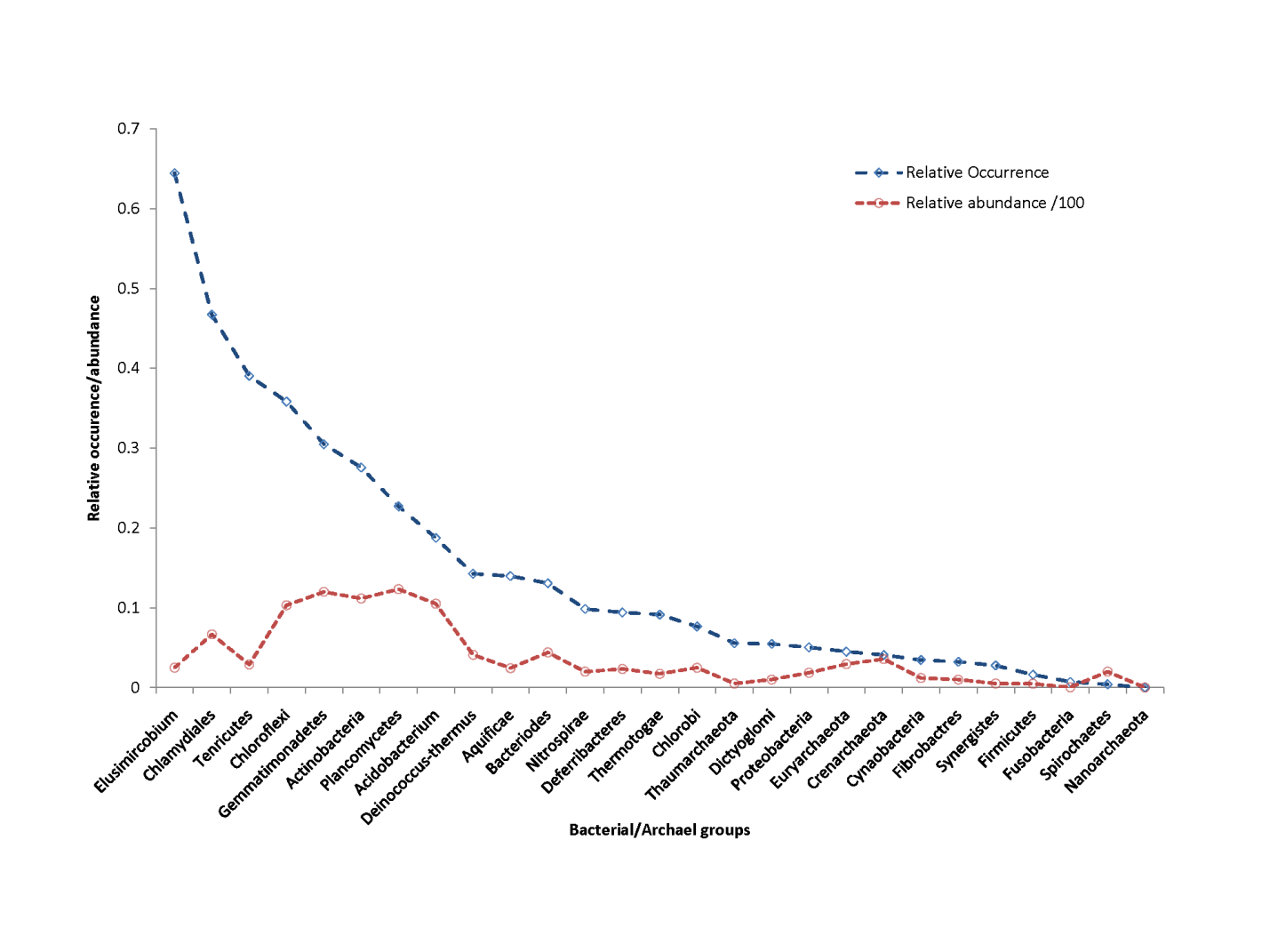


**C. Other domain architectures of POP homologues**

POP homologues in bacterial genomes were also found to co-exist with many other domains e.g. Osmc-like proteins, which are stress induced proteins involved in cellular defense mechanism against oxidative stress caused by exposure to hyperoxides or elevated osmolarity [1, 2]. Most of these were found in phylum *Proteobacteria*. POP domains were also associated with cyclic nucleotide binding domain (proteins that bind cyclic nucleotide), β-lactamase domains (enzymes produced by some bacteria that are responsible for their resistance to β-lactam antibiotics), cellulose-binding domains (associated with enzymes in plant cell wall hydrolysis), FGE-sulfatase (Formylglycine-generating sulfatase that converts newly synthesized inactive sulfatase to their active forms), biotin attachment domain (binds biotin or lipoic acid) and S-layer homology domain (monolayered assemblies of glycol protein which coats the surface of bacteria) [1–8]. The apparent absence of these modules in POP homologues of archaea indicates that they were recruited during evolution for imposing diverse functioning within bPOPs.

**D. Different cellular localization**

Detailed analysis of cytoplasmic and periplasmic POPs was performed in order to understand the differences in sequences which lead to formation of two separate clusters. Interestingly, in cytoplasmic POPs, N-terminal α/β hydrolase domain was very short with a small helix of ~5 residues, whereas periplasmic POPs had large and complete N-terminal α/β hydrolase domain. Most of periplasmic POPs also had a short signal peptide region towards N-terminal. Secondary structure prediction of cytosolic POPs revealed the presence of extra helices at C-terminal α/β hydrolase domain. Some loop and strand insertions were also noticed in periplasmic POPs.

**E. Cluster-wise sequence identity**

To understand how distinct clusters of POPs were formed, average sequence identity of all the clusters was obtained using an in-house MOTIF program. From nine clusters, four clusters had an average sequence identity less than 30%, representing remote relationships between members of the same clusters. Four other clusters indicated direct homology with high sequence identity within the range of 38-80%.


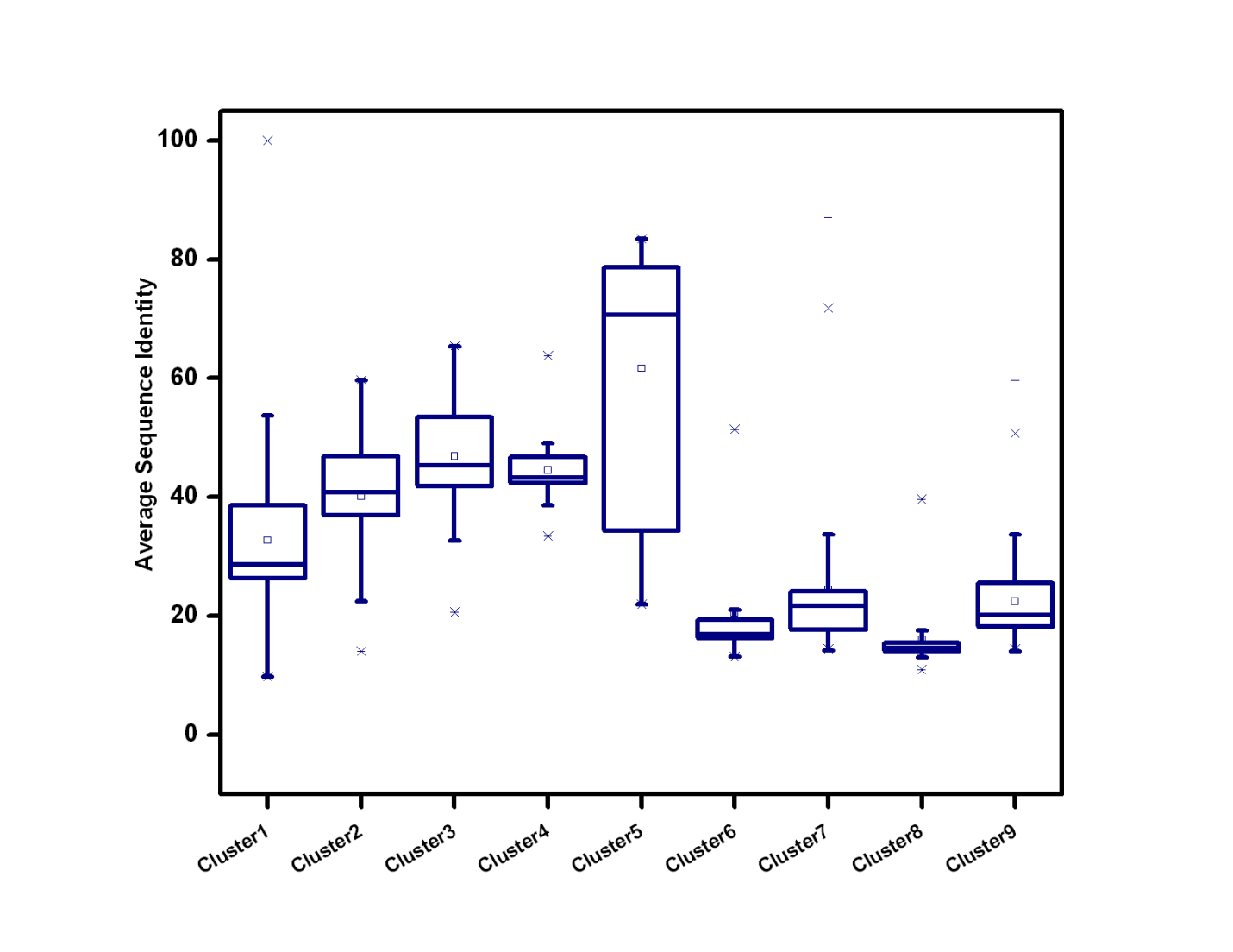


**F. Structural mapping of sequence motifs of each cluster**

In second cluster, two class-specific motifs could be identified. First blade of propeller had surface exposed DQRLYR motif with an average ASA (accessible surface area) of 127Å^2^, while another class-specific motif, PVLLFQG (10.5Å^2^), was located on the hydrolase domain.

Third cluster was associated with high number of class-specific motifs; first and second blade of propeller had NEWOD, GLWRRT, WETLLD and DALAAA, EGENWVW, LSRGGADA motifs, respectively. Loops of third blade possess WIDRDT motif with ASA of 58.66Å^2^, while N-terminal of hydrolase domain was associated with a WVRAQN motif. Three class-specific motifs were located on the blades of propeller (GLQNQSVL, GTVAL, GSDW) and six of them were observed within the hydrolase domain (SKDGTRVPM, WLEMGG, EEWHQAG, VGVLDMLR, DDRVVP, and HSEKI).

Fifth cluster was associated with maximum number of motifs, but only 16 class-specific motifs could be identified. Four of them were solvent exposed with more than 50Å^2^ surface area from which two motifs DERYLK, RFREF were located on N-terminal hydrolase domain and rest were located on fourth and sixth blades of propeller domain. Only one class-specific motif (VGIYGGSYGG) was associated with the catalytic residue Ser563. Motif searches revealed three class-specific motifs in eighth cluster, majorly located on the hydrolase domain of POP. Sequence motif GHSWGGY was associated with catalytic serine.

In ninth cluster, two motifs (NPRGS and GYGQEF) were class-specific in nature, located on a short helix, which was reported to be crucial for interaction with propeller domain through fifth and sixth blades of propeller. NPRGS motif was buried with only 8% ASA, while GYGQEF motif was relatively exposed with 48% ASA.

**G. Functional domains of annotated bacterial POPs are conserved and glycine rich**

Sequence analysis of annotated POPs obtained through exhaustive sequence searches revealed high conservation of catalytic domain (on an average ~44%) as compared to N-terminal α/β hydrolase and β-propeller domains, sharing average sequence identity of 29.63% and 27.22%, respectively. Each cluster showed variations in sequence conservation, for instance, average sequence identity of catalytic domain varied from 24-74%. Highly conserved catalytic domain was present in cluster5, which was mainly rich in POPs of archaebacteria. Residue conservation was calculated to understand the important residues for maintaining the structure and function of the catalytic domain. Structural mapping of functionally important residues obtained through Scorecons revealed their presence near the catalytic site.


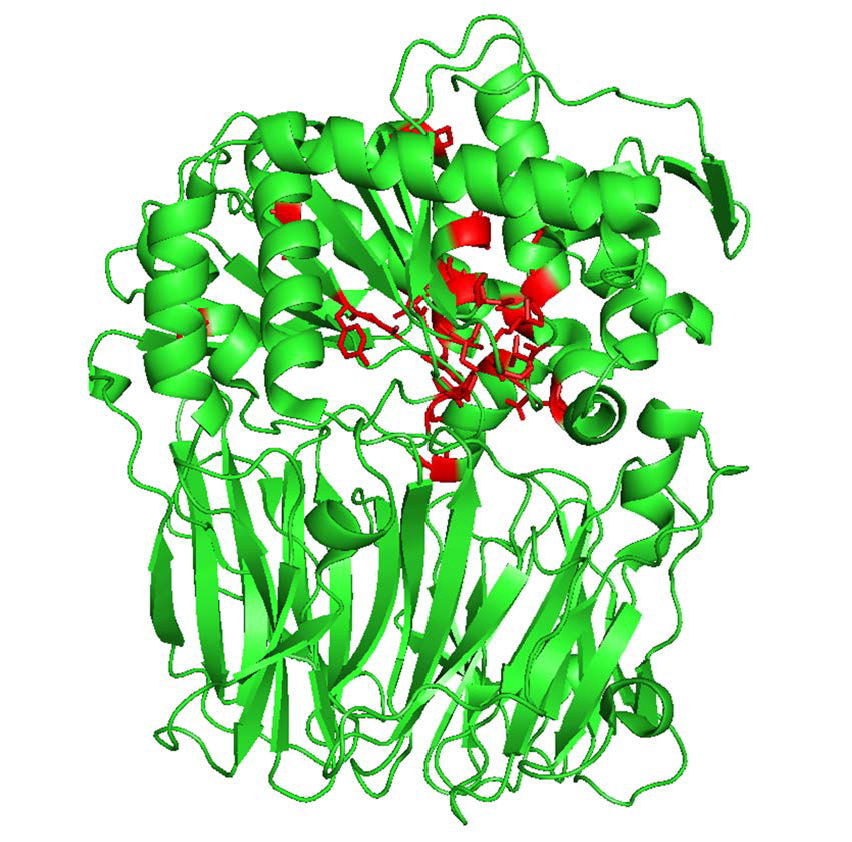


**Figure: Conserved residues mapped on bacterial POP structure (PDB id: 2BKL)**

Conserved residues are colored red.

Interestingly, sequence alignment of annotated POPs from all the domains of life revealed glycine-rich nature of the catalytic domain. Six glycine were found to be highly conserved in POPs (Gly-485, Gly-490, Gly-497, Gly-531, Gly-535 and Gly-536), of which, three of them were present near the catalytic site (Gly-531, Gly-535 and Gly-536), while the other three were located at the interface of catalytic and propeller domains.


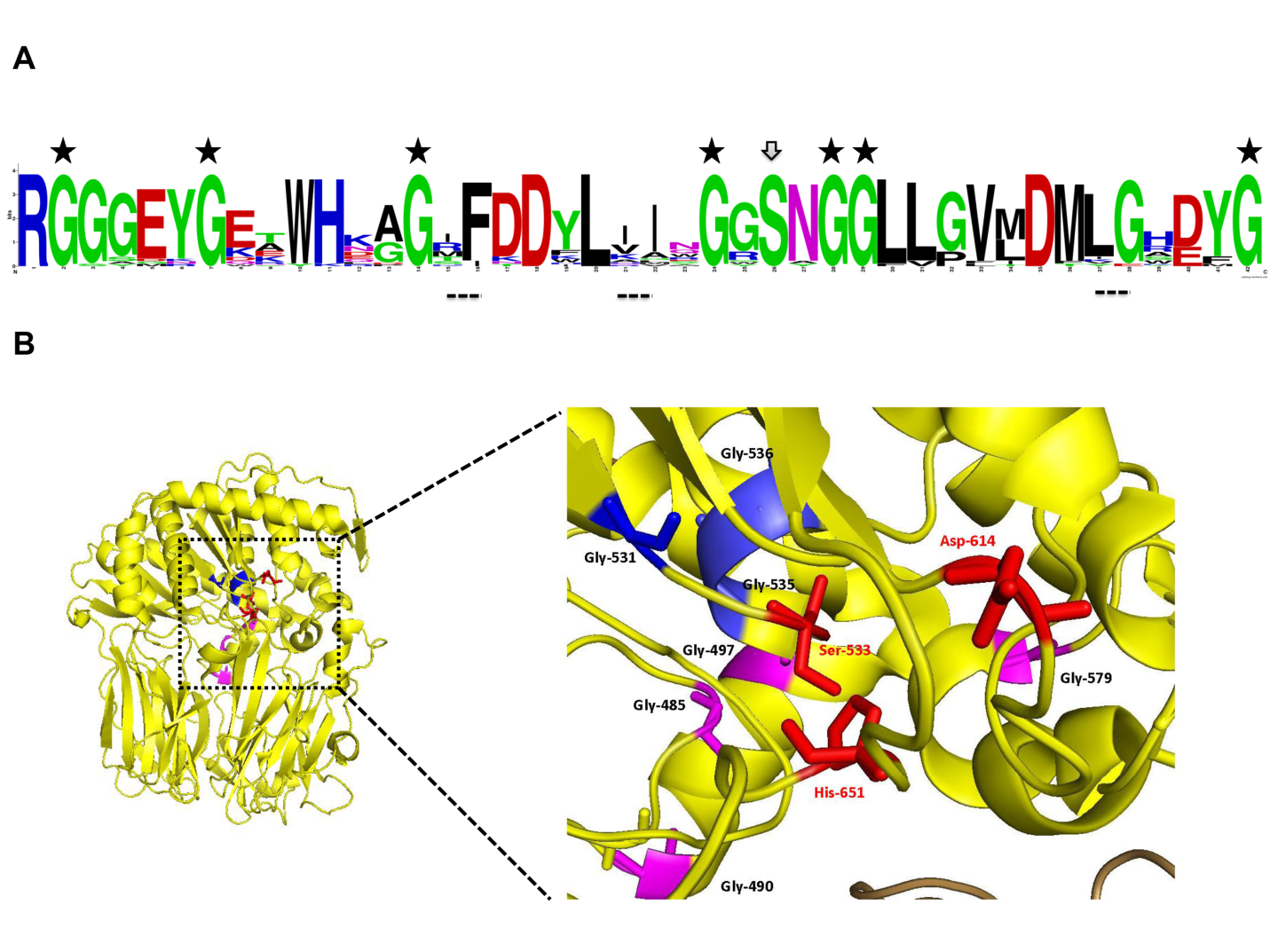


**Figure: Conserved glycine motif in bacterial POPs.**

A) Conserved glycine present in POPs.

B) Structural mapping of conserved glycine on bacterial POP (PDB: 2BKL) from *Myxococcus xanthus*.

**Color code:** Black star represents conserved glycine; catalytic serine is represented by arrow. Black dashed lines represent discontinuity in alignment (only some parts of alignment are shown). Catalytic triad is represented in red sticks; conserved glycine is represented in blue and magenta color (where blue color is for glycine present near active site and magenta for glycine at the interface of two domains of POP (α/β hydrolase and β-propeller)).

**H. Divergence of POP family members**

To test the significance of this co-clustered tree of POP family members, another phylogenetic tree was generated to understand their clustering pattern. In this tree, bPOP sequences were excluded and only DPP, OPB and ACC were considered to obtain better insights about evolution of these members. It was found that in the absence of POPs, other members of this family formed distinct clusters suggesting presence of co-clustering due to POP sequences (data not shown). This indicated similarity of POPs with other members of this family and possibility of their divergence at similar time.

**I. Detailed analysis of POPs of *Shewanella***

Presence of fewer or higher number of POPs in particular phyla or genome can be a consequence of specialization of the organism for a particular proteolytic niche or it is due to acquisition of particular function such as carbon recycling or metal reduction (as in *Shewanella*). In overrepresented POP genomes, catalytic domain was more conserved as compared to the propeller domain. Detailed secondary structure analysis of POPs of *Shewanella woodyi* depicted presence of extra short helices either towards the N-terminal region of α/β hydrolase domain or at the C-terminal domain. Numbers of blades of β-propeller were found to be equal and intact. Sequence alignment of these 16 *Shewanella* POP sequences represented the conservation of catalytic serine and histidine. But, the catalytic triad residue aspartate was less conserved and replaced by small non-polar amino acid glycine or alanine in some of the POP sequences. We suspected that differences in these POPs could possibly due to different cellular localizations. Results of PSORT-b which is a bacterial cellular localization tool indicated that two of these POPs were periplasmic (YP_001761107 and YP_001762910), while one was predicted to be cytoplasmic (YP_001762612) in nature. Difference in cellular localization infers possibility of involvement of POP in different pathways and their sub-functionalization.

**J. Sequence similarity searches to understand HGT events**

Sequence similarity searches were initiated using POPs of these genomes to know how extra copies of POP genes were acquired during evolution. BLAST searches of these POPs (of genus *Shewanella*) against all compiled genomes of bacteria revealed that most of the extra POP genes were gained from other *Shewanella* species. For example, 16 POP genes of *S. woodyi* have possibly been acquired from *S. pealeana, S. baltica, S. frigidimarina, S. halifaxensis, S. sediminis* and *S. violacea* (Additional file 1,Table S1c).

**References**

1. Park S-C, Pham BP, Van Duyet L, Jia B, Lee S, Yu R, Han SW, Yang J-K, Hahm K-S, Cheong G-W: **Structural and functional characterization of osmotically inducible protein C (OsmC) from Thermococcus kodakaraensis KOD1**. *Biochim Biophys Acta* 2008, **1784**:783–788.

2. Macario AJ, Lange M, Ahring BK, Conway de Macario E: **Stress genes and proteins in the archaea**. *Microbiol Mol Biol Rev MMBR* 1999, **63**:923–967, table of contents.

3. Carfi A, Pares S, Duée E, Galleni M, Duez C, Frère JM, Dideberg O: **The 3-D structure of a zinc metallo-beta-lactamase from Bacillus cereus reveals a new type of protein fold.** *EMBO J* 1995, **14**:4914–4921.

4. Carlson BL, Ballister ER, Skordalakes E, King DS, Breidenbach MA, Gilmore SA, Berger JM, Bertozzi CR: **Function and structure of a prokaryotic formylglycine-generating enzyme**. *J Biol Chem* 2008, **283**:20117–20125.

5. Carrard G, Koivula A, Söderlund H, Béguin P: **Cellulose-binding domains promote hydrolysis of different sites on crystalline cellulose**. *Proc Natl Acad Sci U S A* 2000, **97**:10342–10347.

6. Chapman-Smith A, Jr JEC: **Molecular Biology of Biotin Attachment to Proteins**. *J Nutr* 1999, **129**:477S–484S.

7. Esteghlalian AR, Srivastava V, Gilkes NR, Kilburn DG, Warren RA, Saddle JN: **Do cellulose binding domains increase substrate accessibility?** *Appl Biochem Biotechnol* 2001, **91-93**:575–592.

8. Mesnage S, Tosi-Couture E, Mock M, Fouet A: **The S-layer homology domain as a means for anchoring heterologous proteins on the cell surface of Bacillus anthracis**. *J Appl Microbiol* 1999, **87**:256–260.
