# Supplementary material for: Distribution, classification, domain architectures and evolution of prolyl oligopeptidases in prokaryotic lineages
Source: BMC Genomics. 2014 Nov 18;15(1):985. doi: 10.1186/1471-2164-15-985 (PMC4522959; doi:10.1186/1471-2164-15-985)
Supplement: Supplementary file 6 — Additional file 6: Domain architecture of POP homologs. Abbreviations: POP_C-prolyl oligopeptidase C-terminal, DPP_N-Dipeptidyl peptidase N-terminal, WD-WD domain, ABH-α/β hydrolase, DLH-Dienelactone hydrolase, EstPHB-Esterase PHB, Xpro -X-Pro dipeptidyl-peptidase, ABC- ABC transporter, TFB- transcription regulatory domain, TAP- TAP-like protein, TPR- Tetratrico peptide repeats, CNB- cyclicnucleotide binding, Osmc- OsmC-like protein, ASST- Arylsulfo transferase, KAS- beta-ketoacyl synthase, AT- Acyl transferase, AD- Alcohol dehydrogenase, ZnD- Zinc binding dehydrogenase, Branched chain aa- Branched chain amino acid, PRT- Phosphoribosyl transferase, DUF- Domain of unidentified function, BRP- Bacterial regulatory protein, FGS- Formylglycine generating sulfatase, S-layer- S-layer homology, TRD- Transcriptional regulatory domain, SMP-SMP-30/gluconolactonase/LRE-like region. (PDF 432 KB) [file 12864_2014_7072_MOESM6_ESM.pdf]

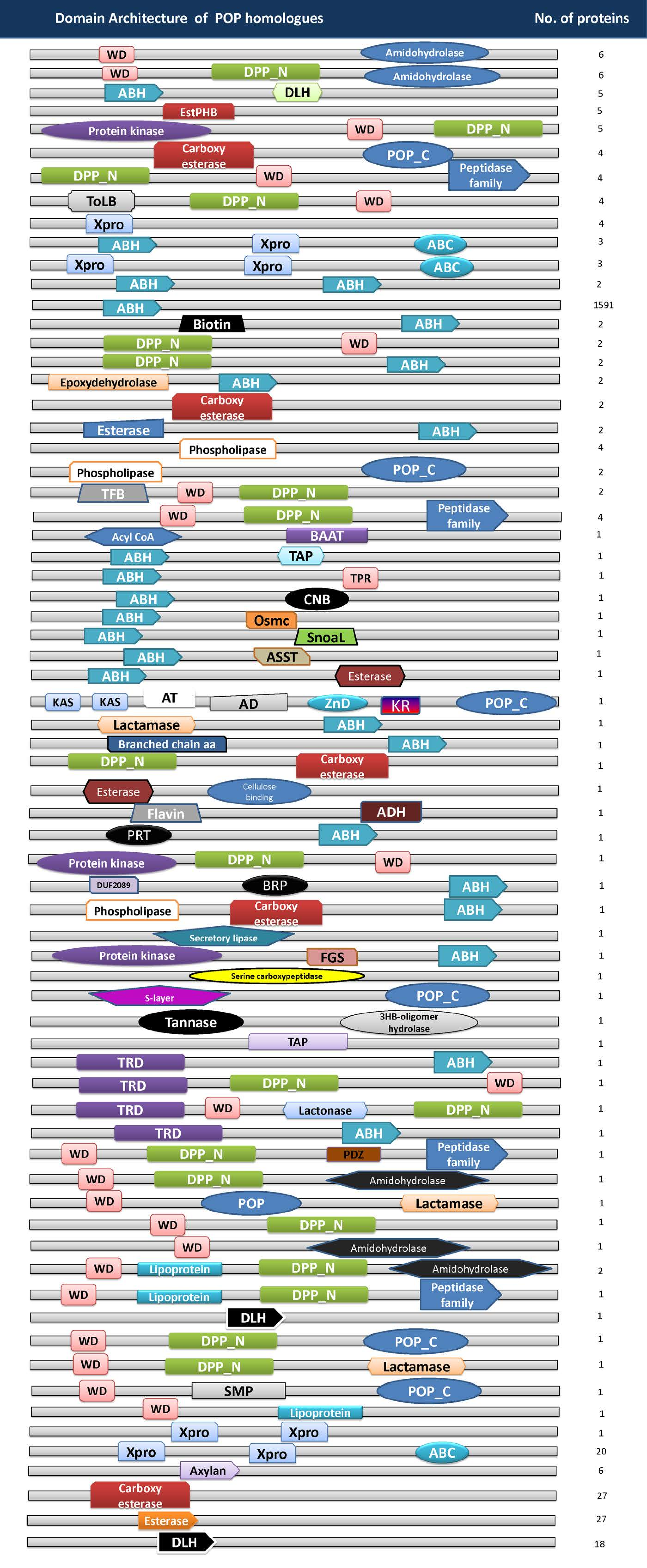
Domain Architecture of POP homologues
No. of proteins
WD Amidohydrolase 6
WD DPP_N Amidohydrolase 6
ABH DLH 5
EstPHB 5
Protein kinase WD DPP_N 5
Carboxy esterase POP_C 4
DPP_N WD Peptidase family 4
ToLB DPP_N WD 4
Xpro 4
ABH Xpro ABC 3
Xpro Xpro ABC 3
ABH ABH 2
ABH 1591
Biotin ABH 2
DPP_N WD 2
DPP_N ABH 2
Epoxydehydrolase ABH 2
Carboxy esterase 2
Esterase ABH 2
Phospholipase 4
Phospholipase POP_C 2
TFB WD DPP_N 2
WD DPP_N Peptidase family 4
Acyl CoA BAAT 1
ABH TAP 1
ABH TPR 1
ABH CNB 1
ABH Osmc 1
ABH SnoaL 1
ABH ASST 1
ABH Esterase 1
KAS KAS AT AD ZnD KR POP_C 1
Lactamase ABH 1
Branched chain aa ABH 1
DPP_N Carboxy esterase 1
Esterase Cellulose binding 1
Flavin ADH 1
PRT ABH 1
Protein kinase DPP_N WD 1
DUF2089 BRP ABH 1
Phospholipase Carboxy esterase ABH 1
Secretory lipase 1
Protein kinase FGS ABH 1
Serine carboxypeptidase 1
S-layer POP_C 1
Tannase 3HB-oligomer hydrolase 1
TAP 1
TRD ABH 1
TRD DPP_N WD 1
TRD WD Lactonase DPP_N 1
TRD ABH 1
WD DPP_N PDZ Peptidase family 1
WD DPP_N Amidohydrolase 1
WD POP Lactamase 1
WD DPP_N 1
WD Amidohydrolase 1
WD Lipoprotein DPP_N Amidohydrolase 2
WD Lipoprotein DPP_N Peptidase family 1
DLH 1
WD DPP_N POP_C 1
WD DPP_N Lactamase 1
WD SMP POP_C 1
WD Lipoprotein 1
Xpro Xpro 1
Xpro Xpro ABC 20
Axylan 6
Carboxy esterase 27
Esterase 27
DLH 18
