# Supplementary material for: Distribution, classification, domain architectures and evolution of prolyl oligopeptidases in prokaryotic lineages
Source: BMC Genomics. 2014 Nov 18;15(1):985. doi: 10.1186/1471-2164-15-985 (PMC4522959; doi:10.1186/1471-2164-15-985)
Supplement: Supplementary file 11 — Additional file 11: A) Mapping of non-permissible amino acid replacement sites on bPOP structure. Color code: Amino acid replacement sites-red, functionally important residues-cyan and green, active site-magenta, catalytic domain-yellow and propeller domain-blue. B) Cluster-wise conservation and replacement of functionally important residues. Top row shows functionally important residues as reported in mammalian POPs. Active site residues are represented in pink. Non-permissible amino acid replacements are shown in yellow. Numbers represent percentage conservation in different clusters. (PDF 345 KB) [file 12864_2014_7072_MOESM11_ESM.pdf]

A

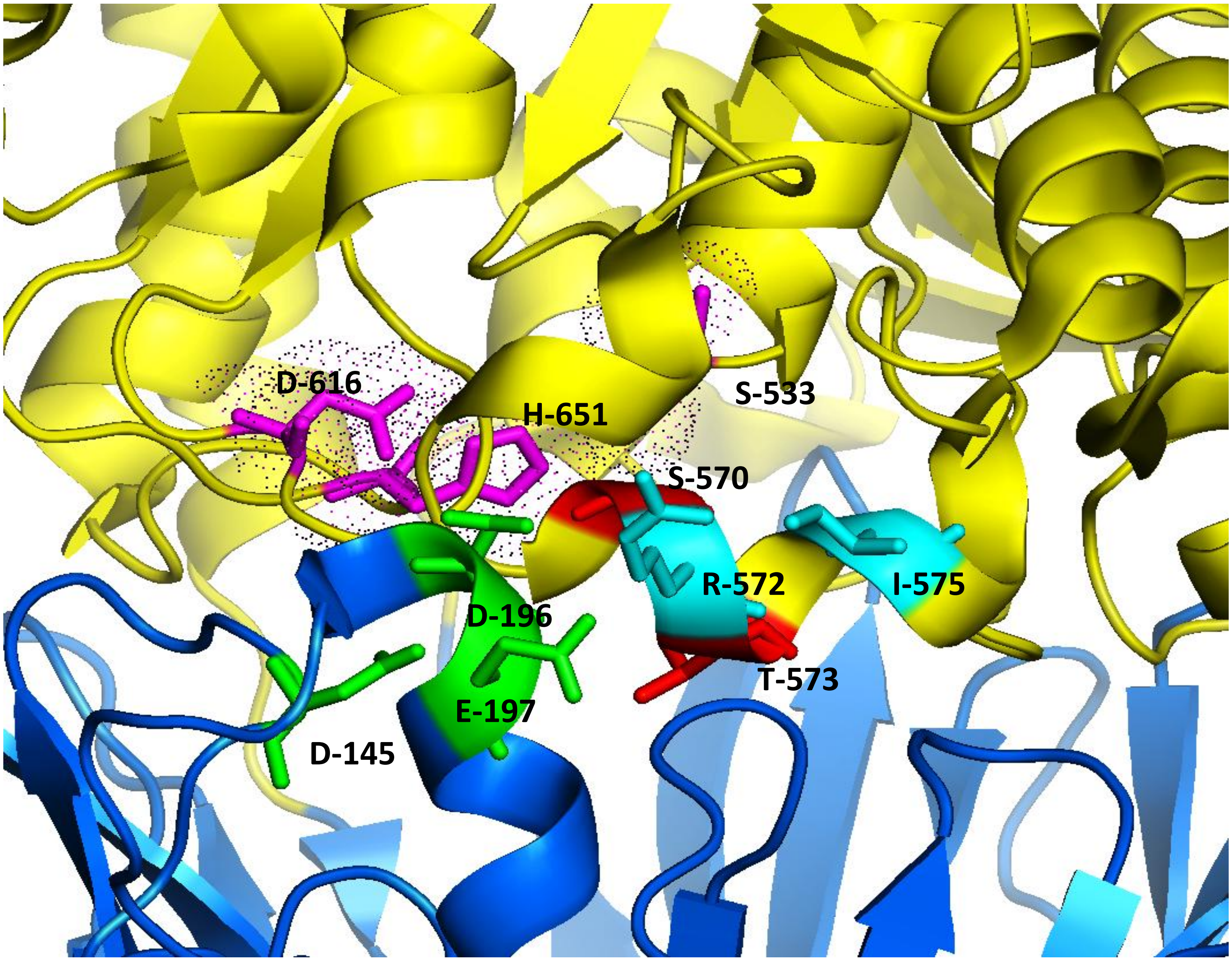

B

	Y-171	F-229	W-249	Y-453	F-456	S-554	N-555	L-560	S-571	T-574	W-575	Y-578	D-641	R-618	V-619	H-680
Cluster1	40	(FY)30	T-40	92	48	92	46	44	L-36	L-46	48	58	92	72	84	92
Cluster2	55	52	84	H-93	P-83	88	A-88	D-93	F-88	R-47	Y-81	L-86	E-40	K-30	54	F-66
Cluster3	95	V-32	L-35	98	95	100	98	91	A-92	S-86	95	98	98	98	95	95
Cluster4	F-87	G-52	T-66	100	100	N-100	V-95	I-75	I-70	A-64	100	97	100	100	100	100
Cluster5	P-72	72	81	100	100	100	100	V-90	I-81	V-72	100	100	100	100	100	100
Cluster6	G-69	D-30	P-26	H-56	P-54	100	Y-67	A-43	L-63	p-76	D-47	76	97	91	97	100
Cluster7	R-24	G-17	T-33	H-100	P-98	98	Y-77	V-43	P-39	R-31	A-20	A-18	99	73	81	63
Cluster8	39	Q-36	Q-48	100	E-24	100	W-54	V-51	A-45	F-48	Q-51	-	I-45	F-42	G-90	100
Cluster9	V-26	D-19	AK-9	H-92	P-98	98	Y-66	S-24	I-33	L-20	23	W-17	100	90	54	96
