# Supplementary material for: Distribution, classification, domain architectures and evolution of prolyl oligopeptidases in prokaryotic lineages
Source: BMC Genomics. 2014 Nov 18;15(1):985. doi: 10.1186/1471-2164-15-985 (PMC4522959; doi:10.1186/1471-2164-15-985)
Supplement: Supplementary file 9 — Additional file 9: Sequence alignment and class specific motifs of each cluster. An arrow represents class specific motifs. (PDF 20 MB) [file 12864_2014_7072_MOESM9_ESM.pdf]

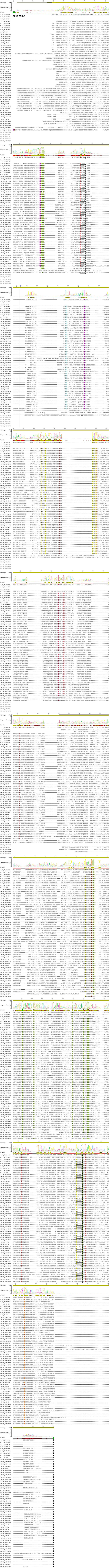
CLUSTER-2
Coverage
Sequence Logo
Identity

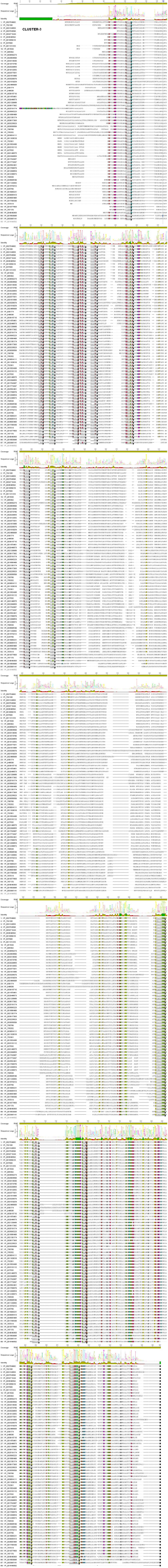
CLUSTER-3

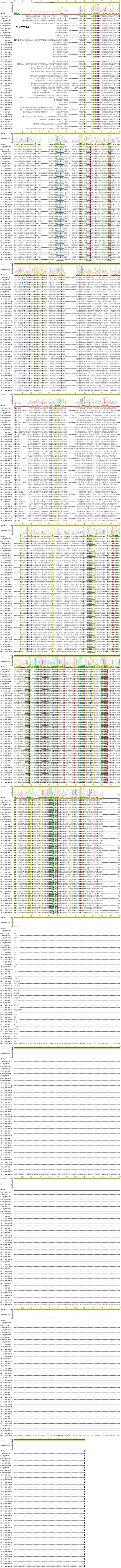
CLUSTER 4

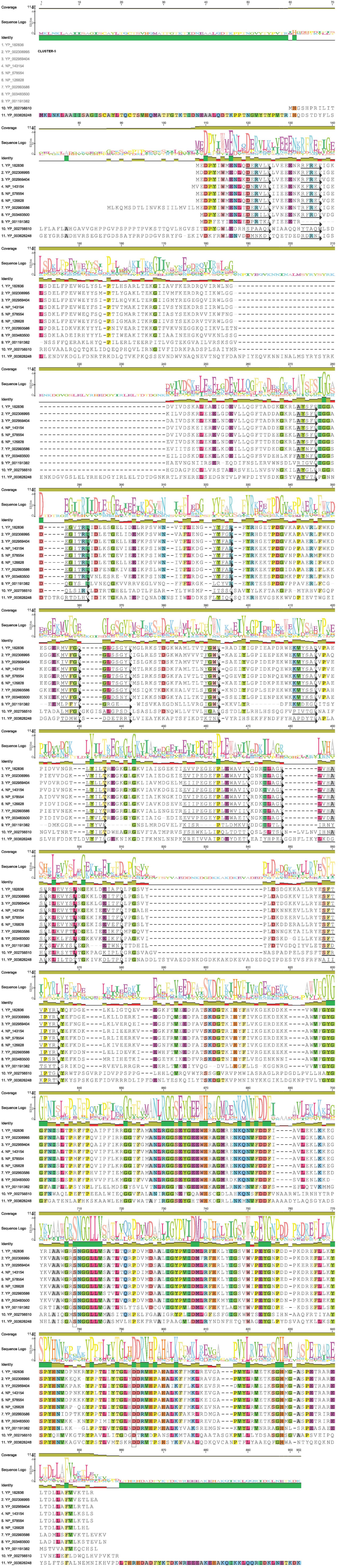
Coverage
Sequence Logo
Identity
CLUSTER-5
1. YP_182836
2. YP_002306995
3. YP_002959404
4. NP_143154
5. NP_578554
6. NP_126828
7. YP_002993586
8. YP_003483500
9. YP_001191382
10. YP_002756510
11. YP_003626248

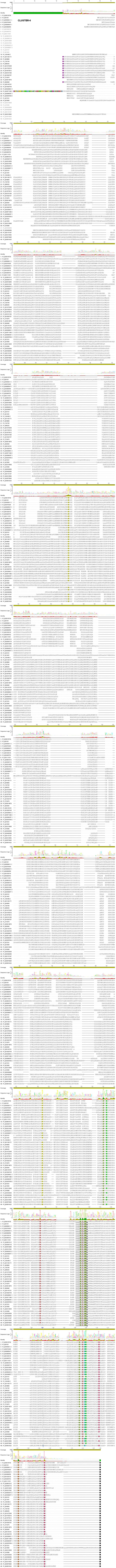
CLUSTER-6

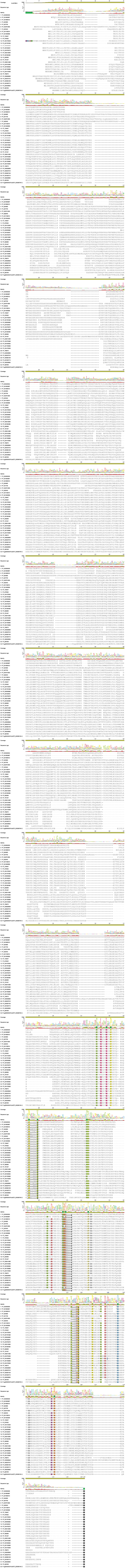
CLUSTER-8
Coverage
Sequence Logo
Identity

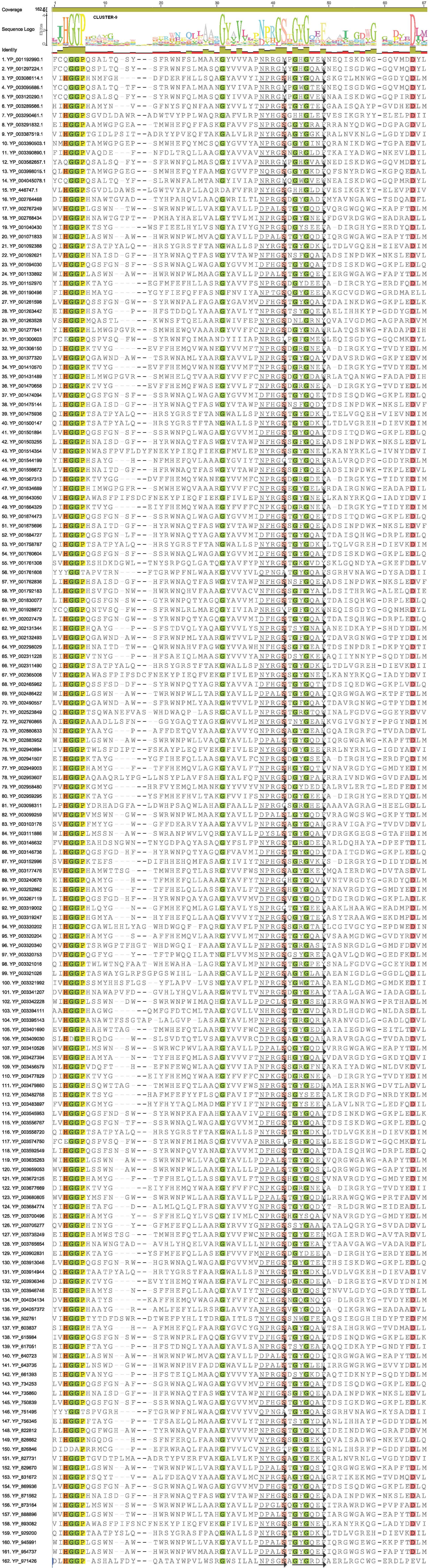
Coverage
Sequence Logo
CLUSTER-9
Identity
